# Incorporating gendered analysis and flexibility in heavy work investment studies: a systematic literature review

**DOI:** 10.3389/fpsyg.2024.1401201

**Published:** 2024-06-19

**Authors:** Carmen Escudero-Guirado, Lourdes Fernández-Rodríguez, Juan-José Nájera-Sánchez

**Affiliations:** ^1^Facultad de Ciencias Económicas y Empresariales, Universidad Pontificia Comillas, Madrid, Spain; ^2^Facultad de Ciencias de la Economía y de la Empresa, Universidad Rey Juan Carlos, Madrid, Spain

**Keywords:** heavy work investment, gender, flexibility, systematic literature review, co-word analysis

## Abstract

**Introduction:**

Significant impacts of heavy work investment on employee well-being and organizational performance have prompted its increasing importance as a research topic. The findings about good or evil of these repercussions are nonetheless inconclusive. The intersection of Heavy Work Investment construct with gender has not been explicitly addressed by previous literature review and research. Besides, the relevance of flexibility for women, as one of the key factors for successful work-family balance management, still remains to be analyzed.

**Methods:**

A literature review on Heavy Work Investment was conducted using the SPAR-4-SLR protocol, wherein 83 articles were selected from a pool of 208 previously identified works. Bibliometric and content analysis techniques were employed, including co-word analysis, to evaluate research production, impact, and trends in the gender perspective within Heavy Work Investment.

**Results:**

As a result, a strategic diagram illustrates thematic topics, providing a clear understanding of the field’s structure and evolution. Six thematic groups were identified, around work-family conflict as the central theme.

**Discussion:**

The explicit consideration of a gender perspective in literature involves nuanced differences regarding the conclusions of studies with a broader focus. First, the emerging prominence of studies on China and Japan becomes clear with gender as the specific focus of the review, aiming to clarify the experiences women face in more traditional societies with a more decisive division of roles. Second, there is a shift in interest regarding the analysis of Job Demands and Job Resources. Despite the apparent decline in interest in the former, the focus in gender literature clearly shifts toward the side of Job Resources, showing potential for the future. It could be understood that in a context of talent war and employee retention efforts, priority is given to better understanding of facilitating individual and organizational factors for work-life balance, especially for women. Future research areas are identified, including gender differences in organizational support and the impact of flexible work on the work-life balance, providing valuable insights for academia, practitioners, and organizations. The need for more comprehensive cross-cultural and gender research is also made clear.

## 1 Introduction

The phenomenon of Heavy Work Investment (HWI) has emerged as a topic of growing importance, since Snir and Harpaz (2012) first defined it as the time and effort invested in work. These authors described it as an umbrella construct, with two main dimensions: time commitment, as in working long hours, and work intensity which implies significant mental and physical effort (Snir and Harpaz, 2012; Tabak et al., 2021). Workaholism and work engagement are recognized as two forms of HWI, with the latter representing the evil, and the former as the good, respectively, (Van Beek et al., 2011; Taris et al., 2015; Schaufeli, 2016). Previous research indicates that work engagement and workaholism are two independent concepts, exhibiting divergent correlation patterns with work-related outcomes; employees characterized by work engagement typically report positive effects, whereas those displaying workaholic behaviors frequently experience negative consequences (Schaufeli et al., 2008; Shimazu et al., 2012; Taris et al., 2015). HWI is conceptualized as a higher-level umbrella construct encompassing various lower-level phenomena including work addiction, work engagement, passion for work, and workaholism, among others (Tziner et al., 2019; Acosta-prado et al., 2021; Tabak et al., 2021).

The interest in researching this phenomenon is justified given its significant impacts on employee well-being and organizational performance, but findings about the good or evil of these repercussions are not conclusive. However, its connection with the physical and mental health of employees is acknowledged. World Health Organization (2022) recognized work-related stress as a “global epidemic,” urging employers and policymakers to promote healthy work environments, by modifying workloads or work schedules to enable work-life prioritization. These negative consequences of HWI also translate into economic costs for organizations and governments. Work-related stress is responsible for over $500 billion in economic costs in the U.S. annually, resulting in the loss of 550 million workdays each year (Moss, 2019). Talented employees have become a powerful source of sustainability and competitive advantage for organizations and being able to retain them is one of the great challenges of management (Mohamad Mazlan and Jambulingam, 2023). Therefore, AN understanding the implications of HWI on work engagement, motivation, job satisfaction, and retirement intentions, among other factors, is relevant.

Numerous studies have examined the antecedents, dimensions, and outcomes of HWI, following the prevalent job demands-resources (JD-R) model (Acosta-prado et al., 2021; Tabak et al., 2021). HWI antecedents previously considered by literature include individual factors, such as personality traits (Hughes and Parkes, 2007; Falco et al., 2014; Mazzetti et al., 2014), individual motivation (Van Beek et al., 2012, 2014; Cooper and Lu, 2019), emotion management (Waghorn and Chant, 2012; Clark et al., 2014; Saleem et al., 2022), and cognitive factors (Van Wijhe et al., 2014). Other perspectives have focused on situational antecedents such as overwork climate in both the family and the organization (Mazzetti et al., 2016; Schaufeli, 2016; Žiedelis et al., 2023), organizational support and organizational policies (Ng and Feldman, 2008; Siu et al., 2010; Costantini et al., 2021), and leadership style and leader support (Ruiz-Palomino et al., 2013; Bowen et al., 2014; Innstrand and Grødal, 2022; Jolly et al., 2022; Dishon-Berkovits et al., 2023; Olsen et al., 2023).

Most of the extensive studies have analyzed separately HWI two distinct dimensions, although some joint research has also been conducted: workaholism (defined as an addictive attachment to work) and work engagement (something that denotes a favorable connection to and immersion in one’s work). Huml et al. (2020) examine the relationship between work engagement and workaholism among employees in the sports industry, alongside various work-related and individual factors, which could both encourage or mitigate workaholism, such as job flexibility and gender. Their findings indicated that women were more likely to report higher levels of workaholism than men, without higher levels of work–family conflict; rather, it was men who reported significantly higher levels. Authors suggests that men may experience higher levels of conflict than women, because social norms are evolving toward greater paternal involvement at home. Additionally, men reported higher levels of work engagement compared to women. While these findings align with previous research, authors claim for a more nuanced investigation and theorization to understand the gender differences in reported work engagement and workaholism. Di Stefano and Gaudiino (2019) meta-analyze available studies on the relations between subdimensions of workaholism and work engagement and conclude that both concepts overlap and are moderated by nationality.

This work considers gender only as a potential moderator of workaholism and work engagement relationship and concludes that gender did not significantly affect. Andersen et al. (2023) present a first meta-analysis and systematic review of workaholism prevalence in 23 countries, including a discussion on the concept and its measurement, but without any reference to gender differences. Cheng and Gu (2022) explore the relationship between workaholism and work performance by meta-analysis and demonstrate that workaholism and its dimensions (working excessively and working compulsively) exert varying effects on distinct facets of work achievement. This article identifies gender differences only as a future research direction. Russo et al. (2023) test the workaholism-personal burnout relationship in a sample of dual-career couples. Their findings highlight the complex interplay between gender roles, family dynamics, and the consequences of workaholism. However, according to the authors, the small sample and the need to consider other types of couples encourage further research in this direction.

In addition to these, many other studies have examined workaholism, its antecedents, and consequences in one way or another (Bakker et al., 2009; Shimazu et al., 2011; Xu and Li, 2021; Ayar et al., 2022; Eason et al., 2022; Falco et al., 2022). In relation to work engagement, Cheng et al. (2022) confirm that unreasonable tasks negatively impact work engagement by activating cognitive and affective responses, however supervisor support can mitigate these effects. Other studies have analyzed work engagement as a mediator between job resources and work-family enrichment (Siu et al., 2010; Hakanen et al., 2011) or as affected by work–family conflict (Lyu and Fan, 2022; Chen et al., 2023; Lu, 2023; Tang et al., 2023).

Finally, regarding the consequences of HWI, prior literature has examined individual consequences such as effects on health (both physical and mental) (Kato and Yamazaki, 2009; Carlson et al., 2011; Milner et al., 2017; Pien et al., 2021; Lange and Kayser, 2022; Milicev et al., 2023), work–family conflict (Cinamon and Rich, 2010; Lee et al., 2021; Eason et al., 2022; Ratnaningsih et al., 2023; Tang et al., 2023), burnout (Houkes et al., 2008; Dishon-Berkovits, 2014; Beauregard et al., 2018; Pujol-Cols, 2021; De Beer et al., 2023; Russo et al., 2023), stress (Demerouti et al., 2005; Choi, 2008; Bowen et al., 2014; Nohe et al., 2015; Oshio et al., 2017; Olsen et al., 2023), intention to leave (Dåderman and Basinska, 2016; Minamizono et al., 2019; Rajendran et al., 2020; Jolly et al., 2022), job and family satisfaction (Demerouti et al., 2005; Bakker et al., 2009; Hakanen et al., 2011; Syrek et al., 2022), and well-being (Hughes and Parkes, 2007; Shimazu et al., 2011; Eek and Axmon, 2013; El-Kot et al., 2019), among others. Job performance (Shimazu et al., 2012; Falco et al., 2013; Shimazu et al., 2015), affective or aggressive behaviors (Tricahyadinata et al., 2020) and organizational citizenship behaviors (Choi, 2013; Singh and Banerji, 2022; Dеу, 2023; Mer et al., 2023) are identified as HWI situational outcomes.

Much of this research has addressed the specificity of certain professional groups that are particularly prone to dedicating more time and work demanding than others, such as healthcare professionals – doctors and nurses (Day and Chamberlain, 2006; Pal and Øystein Saksvik, 2006; Ádám et al., 2008; Houkes et al., 2008; Estryn-Behar et al., 2011; Lu et al., 2011; Milner et al., 2017; Minamizono et al., 2019; Zhang et al., 2020; Abusanad et al., 2021; Lee et al., 2021; Ayar et al., 2022; Bradfield et al., 2022); teachers (Rajendran et al., 2020; Tang et al., 2023) and sport professionals (Eason et al., 2014, 2022; Mazerolle et al., 2015, 2018; Huml et al., 2020), to name just a few.

### 1.1 The presence of gender in HWI literature

The literature has considered from various perspectives the impact of gender on the antecedents, dimensions, and consequences of HWI, with inconsistent results concerning both the strength and direction of the connections among them. Considering this construct as a continuum in accordance with current theoretical proposals that use the JD-R model (Tabak et al., 2021), for the purposes of this work it is assumed that findings relating gender to any of these three parts can be extended to the construct of HWI.

This section presents specific findings regarding the relationship of gender with some antecedents (such as job routinization and organizational aspects like flexibility) and outcomes (such as stress, emotional exhaustion, work-family interference, or burnout) of HWI. For instance, Roxburgh (1996) analyzes the impact of job stressors on both women and men well-being and concludes that job demands have conditional effects: higher distress in women is not solely attributable to job stressor exposure, since they are more vulnerable to the negative effects of job routinization. Intending to clarify the mixed results of previous research, the findings of Purvanova and Muros (2010) go against the commonplace idea that women face burnout more than men and show that female individuals experience slightly higher emotional exhaustion when compared to male ones, with men tending to exhibit somewhat greater depersonalization. In their studies on the differences in the use of working and family time, Mattingly and Bianchi (2003) findings suggest that men and women have distinct experiences with leisure time, and they feel the consequences of work-family interference differently. Men experience greater benefits as they compartmentalize their activities and concerns between work and family life, and this may be influenced by socialization and interconnected work-family experiences.

The literature suggests gender is one of the most significant variables, since it touches upon affecting the different experience of working at home, the features, and outcomes of flexible work arrangements, as well as its efficiency. For instance, Field et al. (2023) conclude that women continue to value flexibility more than men, because they still carry out a disproportionate amount of childcare and household work. Indeed, 38 percent of mothers with young children say in this report that without workplace flexibility, they would have had to leave their company or reduce their work hours. According to Powell and Craig (2015) women may be more likely to work at home to accommodate work and family demands, while men would make that choice to facilitate additional employment time. Wall and Arnold (2007) findings propose that, despite the advances in co-parenting, women maintain their position as primary parents and are, therefore, more susceptible to suffering the negative effects of Work Interference with Family Life (WIF). Borelli et al. (2017) agree in pointing out that mothers experience more conflict and guilt than fathers because of WIF. Despite the complex and sometimes contradictory findings regarding gender differences in the interaction between work and family, overall, the available empirical evidence suggests that it has a more profound impact on women than on men (Innstrand et al., 2009). While some studies report that women experience higher levels and frequencies of work–family conflict (negative outcome of HWI) (Cinamon and Rich, 2002; Hill et al., 2004), others suggest that gender has only a limited moderating effect on work-family balance (Aryee et al., 2005; Kinnunenn et al., 2006) or find no significant impact of gender at all (Ford et al., 2007). However, research into gender differences in facilitation, such as positive spillover from work to family and positive outcome of HWI, is largely unexplored. This highlights a critical area for further research to design targeted interventions to support women in managing these dual roles.

While previous literature reviews have been conducted on the concept of HWI, none of them have explicitly addressed the intersection of this construct with the gender perspective and the relevance of flexibility for women, as one of the key factors for successfully managing the impact of HWI on WIF. WIF has been the dominant perspective in these previous works, whether from a global perspective (Allen and Martin, 2017; Reimann et al., 2022) or with a focus on an additional issue [such as the impact of the COVID-19 pandemic in the case of Vitória et al. (2022); a life and career stage perspective as in Demerouti et al. (2012); or its effect on job satisfaction in Kong et al. (2018)].

In this context, this paper contributes to the advance of research on HWI by incorporating a specific focus on gender and the relevance of flexibility to manage the WIF and providing: (i) Characterization of the literature in this specific field, considering the evolution of production and papers impact, authors and their origins, journals, main cited articles (intellectual foundation), and topics addressed; (ii) Conceptual structure of the domain, establishing relationships between topics and grouping them according to their affinity through co-word analysis; (iii) Study of the characteristics of these groups, in terms of size, currency, impact, density (degree of internal cohesion of the topics), and centrality (importance for the development of the topic); (iv) Predictable evolution of thematic clusters; and (v) Identification of future research avenues related to each group.

Finally, our work offers valuable insights on how gender, work-family dynamics, and flexibility intersect. On one hand, it identifies the growing potential of research around job resources, and more specifically, around organizational factors, as determinants for work-life balance, and ultimately for women’s talent retention and promotion. On the other hand, it offers substantial contributions, benefiting academia, as well as practitioners and organizations. In terms of academia, our findings emphasize the need for methodological innovation beyond the limitations of traditional cross-sectional studies. There is also potential for enhancing the measures, that currently rely heavily on self-reports and perceptions. By incorporating a more nuanced exploration of gender (viewed as a continuum of masculine and feminine behaviors) and conducting cross-cultural studies, we can achieve more definitive conclusions about HWI and gender. For practitioners and organizations, we find evidence to support not only the implementation but also the need for appropriate measurement of most effective practices (such as autonomy, career development encouragement, diversity recognition, systemic support and effective team leaders and HR managers), while acknowledging gender diversity.

## 2 Method

**Figure 1:**
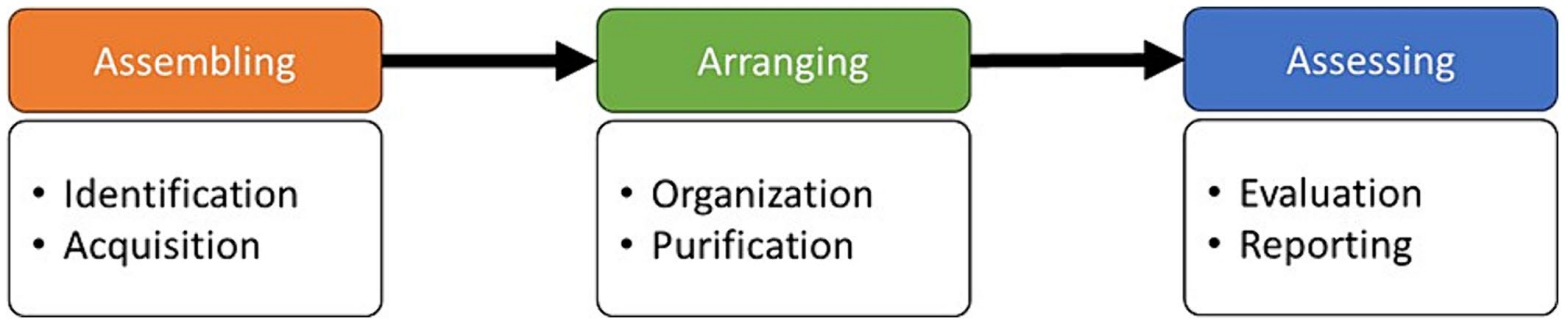
The SPAR-4-SLR protocol.

To address the research questions posed, we conducted a systematic literature review following the SPAR-4-SLR protocol (Paul et al., 2021). We adopted this protocol instead of PRISMA or PRISMA-P (Moher et al., 2009, 2015) because these protocols were developed for systematic reviews in general while SPAR-4-SLR is adapted to social science fields, providing rationales to justify the decision-making in the review. This protocol consists of six steps distributed across three stages (see Figure 1).

### 2.1 Assembling and arranging

Within the Assembling phase, the first step is called Identification. In the introduction of this work, we have already defined both the domain and the research questions. In our review, we have included exclusively articles published in academic journals. We have excluded other documents (such as books, proceedings, and other editorial materials) because we aim to conduct a representative review, not necessarily an exhaustive one. Podsakoff et al. (2005) demonstrate the high concentration of citations in fewer academic journals, which justifies our decision. Finally, given the specificity of the topic, we decided to establish the sole quality criterion for publications as their inclusion in the Science Citation Index Extended (SCI) and the Social Science Citation Index (SSCI).

Regarding Acquisition, the second step in this initial phase, we have chosen the Web of Science (WoS) search engine. This database offers several advantages to performing this phase. First, it allows the selection of publications included in SCI and SSCI exclusively. Second, it is possible to download some of the necessary metadata to perform the research. We only excluded 2024 from our search and did not establish additional limits to the period, keeping in mind the specificity of the topic. Based on previous systematic reviews on similar topics (Acosta-prado et al., 2021), we run the following query in February 2024: ts = (“heavy work investment” OR workaholism OR “work addiction” OR “passion to work” OR “job demands” OR “work craving” OR “work engagement” OR “addiction to work” OR “passion toward work” OR “passion for work” OR “heavy-work investment”) AND ts = (“work-life balance” OR “job flexibility” OR “work flexibility” OR “flexibility at work” OR wlb OR “work life balance” OR “work-life-balance” OR wfc OR “work-to-family conflict” OR “work–family conflict” OR “family–work conflict” OR wfb OR “work-family balance” OR wfe OR “work-family enrichment” OR “family-work balance” OR “family-work enrichment”) AND ts = (gender OR femen* OR femal* OR wom?n OR glass ceiling OR diversity).

After defining our research domain, we have included three different components in this query. The first one gathers all the relevant keywords in the heavy-work investment arena. The second focuses on the work flexibility line. The third comprises the different terms used in the literature to refer to gender issues. We limited our search to documents written in English. This query returned 208 articles.

The first step of the arranging phase, Organization, implies the codification of the documents. Considering the bibliometric nature of this systematic review, we obtained the data from the WoS database, codifying the information about publication year, title, outlet, abstract, author keywords, citations, and authors (including affiliation) for each document. With this information, two researchers did a manual screening independently, reading title, abstract and keywords to check if the articles should be included in the final sample. In case of doubt, both researchers read the full article and discussed the criteria. Sixty-seven articles were excluded because they did not deal with HWI-related topics, four because they did not address any topic related to flexibility, 16 because gender was not a relevant issue, and 38 because a combination of these three motives (35 of them lacked HWI-related topic, and five did not address the three conditions)—the final sample comprised 83 articles.

### 2.2 Assessing

In the evaluation step, we used a combination of bibliometric techniques and content analysis. Regarding the bibliometric study, we have analyzed some measures concerning the production and impact of this research line, the main outlets, and the authors’ origin. These analyses allow us to answer the questions related to the evolution of the topic and characterize it.

The second analysis is based on the co-word technique. This examination identifies relationships between subjects in a research field and helps tracing a research domain’s content structure and evolution (He, 1999). Callon et al. (1983) were the first to propose the co-word analysis method to identify and represent associations between concepts from textual information. The logic behind this method is straightforward: if the same document analyzes two topics, it is because of a relationship between them. The study of the co-occurrences between topics in the documents included in a research domain allows for mapping its conceptual structure and drawing the relationships among them in a semantic map (Zupic and Čater, 2015).

This technique requires to make some decisions prior to the analysis. First, it is necessary to assign topics to the documents. The most frequent options are using fields like author keywords or KeywordPlus(r). However, this alternative involves several limitations to consider. To begin with, there is no standardization in these terms, so a previous treatment of these keywords is necessary. Choi et al. (2011) suggested some helpful rules to complete this task. They advise standardization into unique forms (e.g., turnover intent and turnover intention), the consolidation of a term and its possible abbreviatures (e.g., WFC and work–family conflict), the unification of synonyms (e.g., workaholism and addiction to work) and the separation of compound terms (e.g., African American women and the workplace), and the elimination of terms without a clear meaning or too a general one (e.g., framework).

Furthermore, we removed those terms related to location and methods because of the focus on the content related to the analyzed issues. Besides, three researchers screened the list of author keywords for each article and read the abstract to verify if the words were representative. Additional keywords were suggested for articles that needed more than four (less than four) and those without any. The additions were discussed and only introduced when there was agreement.

To support all these processes and build the final network, we used Bibexcel software (Persson et al., 2009) and analyzed with VOSViewer (Van Eck and Waltman, 2010). It includes functionalities to normalize the relationships and cluster and map the terms. Although there are many options for normalization, Van Eck and Waltman (2009) showed the advantages of association strength, comparing it to other popular choices, like Salton’s cosine. These authors also have defended the accuracy of the VOS algorithm for clustering and mapping (Van Eck and Waltman, 2014) and the advantages of combining both methods. Thus, we have adopted these options for our analysis, following recent research (e.g., Mora-Valentín et al., 2022).

To complete the analysis, we have calculated Callon’s centrality and density for the clusters obtained to represent the thematic groups in a strategic diagram (Callon et al., 1991). The centrality of a cluster is calculated as the weighted degree of the nodes included in a cluster regarding the nodes in the rest of the network. It represents the importance of that cluster for developing a research field. The density of a cluster is the average weighted degree of the nodes of a cluster only regarding that cluster. It shows how strong the relationships between the topics included in a group are. According to these two dimensions, the strategic diagram allows the identification of four kinds of topics: motor themes, with a high centrality and density; basic and transversal themes, with a high centrality and low density; highly developed and isolated themes (low centrality, high density); and emerging and declining themes (low centrality, low density).

**Figure 2:**
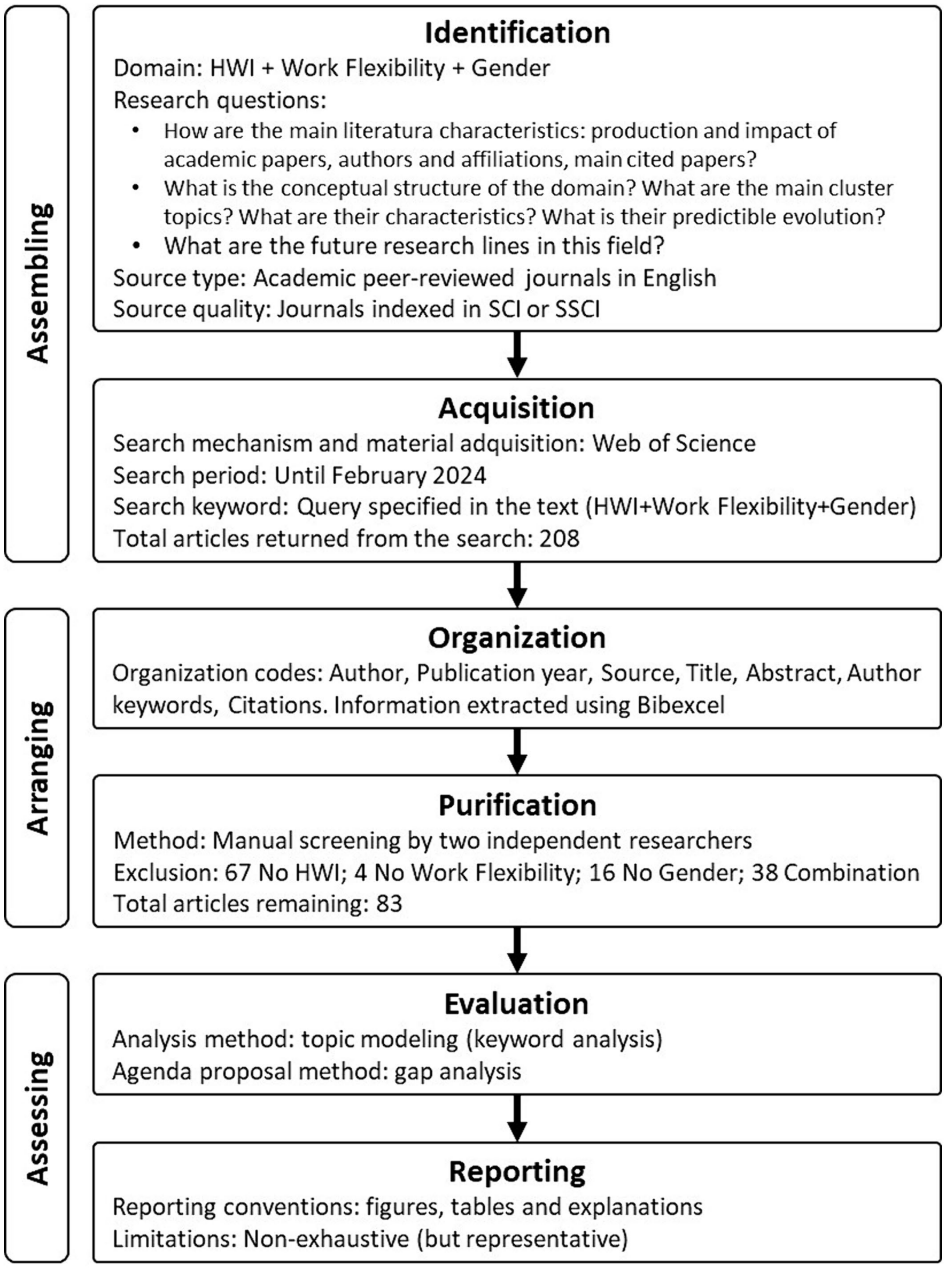
SPAR-4-SLR diagram of systematic review process.

Figure 2 summarizes the methodological process with the SPAR-4-SLR diagram of systematic review.

## 3 Results

### 3.1 The gender perspective in research on HWI: evolution and trends

**Figure 3:**
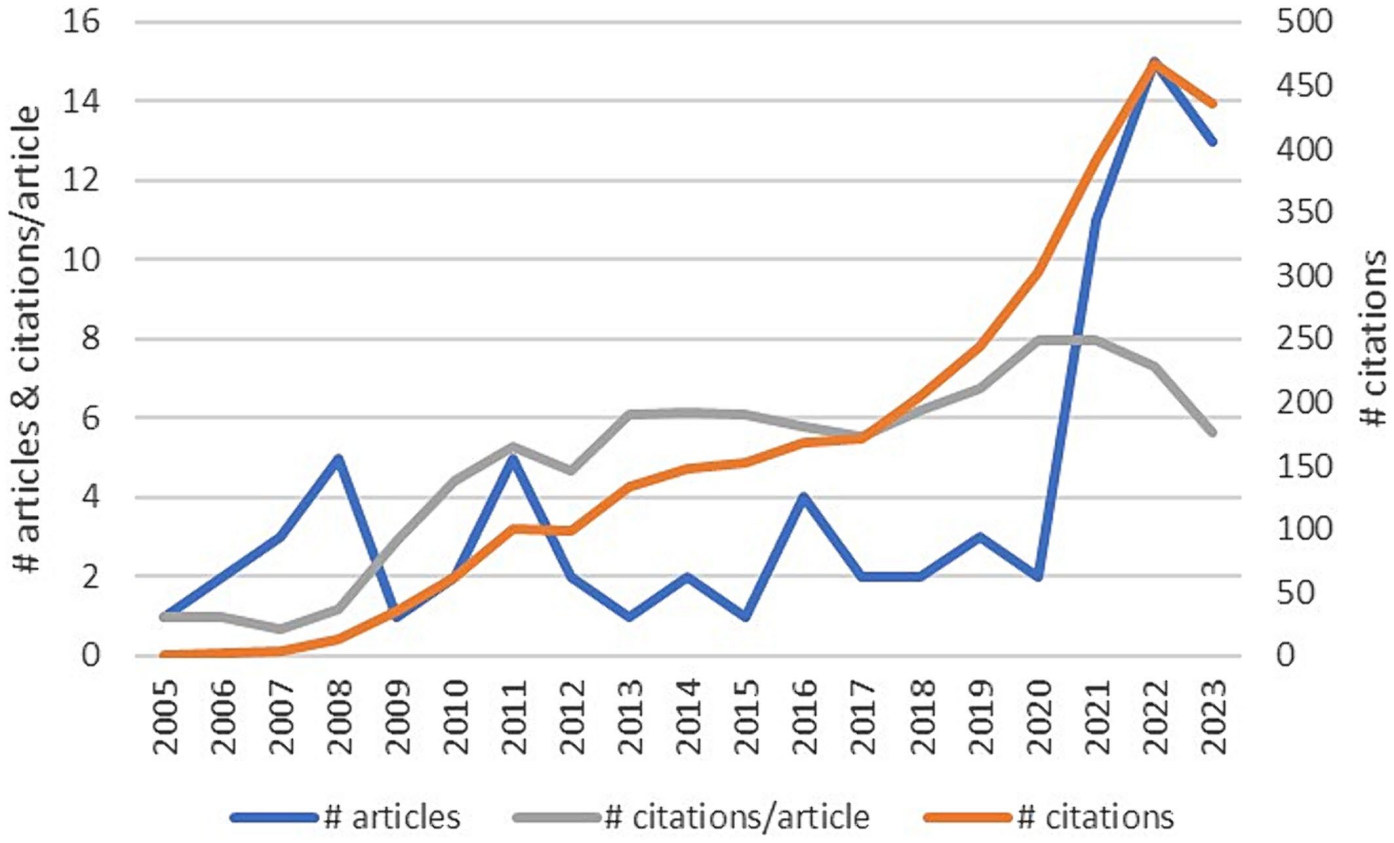
The evolution of the domain.

Figure 3 shows the evolution of this research line. Regarding production, we can see that since 2005, the publication of articles dealing with this topic has been interrupted. Although the figures have been erratic, last 3 years production of more than 10 articles each might be the promise of an expanding interest in this domain. Similarly, we can observe the exponential growth of the number of citations per year and the number of citations per year and article, confirming the previous conclusion.

Two hundred eighty-five different authors have written this production, most of them with only one contribution (94.03%) and only three with three or more articles: Demerouti (5) and Bakker (5) focus on spillover-crossover effect, and Burke (3) who deals with well-being related themes. Analyzing the origin of this production, we can observe that only four countries (China, Canada, EEUU, and the Netherlands) have more than 10 contributions. The production of Australia (8), Japan (5), and some European countries, especially from the north of Europe (Sweden, Norway, Finland, Germany, United Kingdom) and Italy, is also remarkable. Finally, we have analyzed the collaboration. Only six articles have been authored by one researcher. The most frequent number of contributors has been three (23 papers). Of the 77 articles with more than one author, 43 have authors only from one country, and 24 of them have researchers from two countries. The Netherlands (9 papers) and China (8 papers) were the most internationally collaborative countries, along with Canada (5) and Australia (5).

The most frequently cited research was Greenhaus and Beutell (1985), one of the seminal studies about work–family conflict. It was quoted by almost 50 % of the articles in our database. The work of Netemeyer et al. (1996) containing the scales for family–work conflict and work–family conflict was mentioned in 22 works, and Schaufeli and Bakker (2004), about job demands, job resources and their relationship to burnout and work engagement was referenced in 20 papers. It is also remarkable that this list includes the work of Hobfoll (1989), the origin of the Conservation of Resources theory. Table 1 gathers some details of the works with 10 or more citations in our database (excluding general methodological references).

**Table 1:** Most cited articles.

Authors	Year	Title	Citations
Greenhaus, JH; Beutell, NJ	1985	Sources of conflict between work and family roles	41
Netemeyer, RG; Boles, JS; McMurrian, R	1996	Development and validation of work–family conflict and family–work conflict scales	22
Schaufeli, WB; Bakker, AB	2004	Job demands, job resources, and their relationship with burnout and engagement: a multi-sample study	20
Demerouti, E; Bakker, AB; Nachreiner, F; Schaufeli, WB	2001	The job demands-resources model of burnout	20
Hobfoll, SE	1989	Conservation of resources – A new attempt at conceptualizing stress	19
Bakker, AB; Demerouti, E	2007	The Job Demands-Resources model: state of the art	18
Byron, K	2005	A meta-analytic review of work–family conflict and its antecedents	17
Grzywacz, JG; Marks, NF	2000	Reconceptualizing the work-family interface: an ecological perspective on the correlates of positive and negative spillover between work and family.	17
Allen, TD; Herst, DE; Bruck, CS; Sutton, M	2000	Consequences associated with work-to-family conflict: a review and agenda for future research.	16
Edwards, JR; Rothbard, NP	2000	Mechanisms linking work and family: Clarifying the relationship between work and family constructs	12
Karasek, RA	1979	Job demands, job decision latitude, and mental strain – implications for job redesign	12
Frone, MR	2003	Work-family balance	12
Gutek, BA; Searle, S; Klepa, L	1991	Rational versus gender-role explanations for work family conflict	11
Schaufeli, WB; Bakker, AB; Salanova, M	2006	The measurement of work engagement with a short questionnaire – A cross-national study	11
Frone, MR; Russell, M; Cooper, ML	1992	Antecedents and outcomes of work family conflict – Testing a model of the work family interface	11
Greenhaus, JH; Powell, GN	2006	When work and family are allies: A theory of work-family enrichment	11
Frone, MR; Yardley, JK; Markel, KS	1997	Developing and testing an integrative model of the work-family interface	11
Maslach, C; Schaufeli, WB; Leiter, MP	2001	Job burnout	10
Carlson, DS; Kacmar, KM; Williams, LJ	2000	Construction and initial validation of a multidimensional measure of work–family conflict	10
Schaufeli, WB; Salanova, M; Gonzalez-Roma, V; Bakker, AB	2002	The measurement of engagement and burnout: a two-sample confirmatory factor analytic approach	10
Eby, LT; Casper, WJ; Lockwood, A; Bordeaux, C; Brinley, A	2005	Work and family research in IO/OB: Content analysis and review of the literature (1980-2002)	10

Source: Own elaboration.

Finally, in regard to the outlets in our database, the articles were published in 52 different publications, which suggests a very dispersed literature. Only 14 journals contain more than one article, and just three of them have published five or more works: International Journal of Environmental Research and Public Health, Journal of Vocational Behavior and Frontiers in Psychology. Analyzing the research areas, we found that 37 of the 52 were included in Psychology, 19 in Business & Economics, and Public, Environmental & Occupational Health.

### 3.2 Co-word analysis

**Figure 4:**
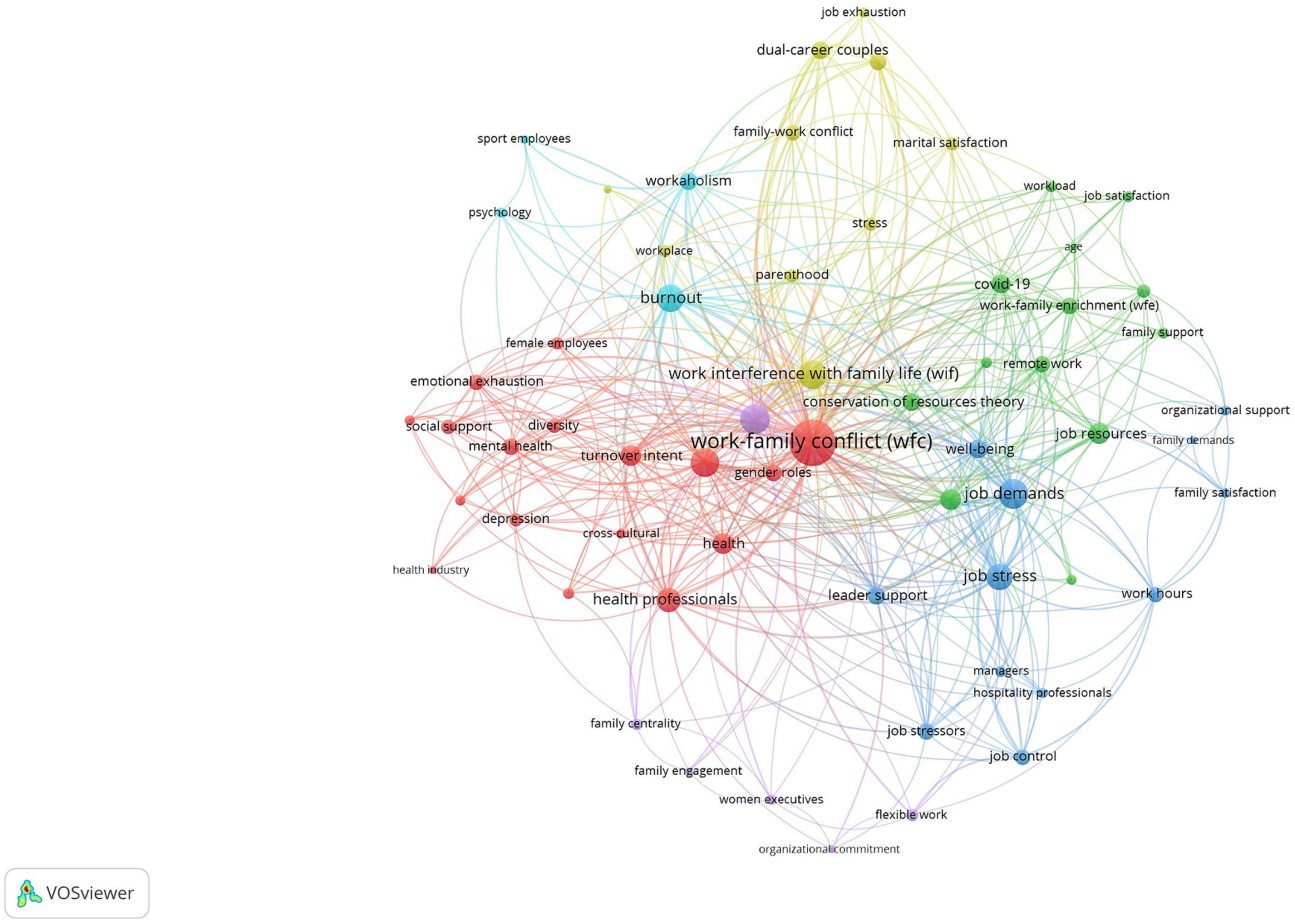
Co-word network.

After a curating process, 124 terms were kept in our database. Table 2 contains the most frequent keywords. We performed the co-word analysis using exclusively those keywords appearing twice or more in the database. Considering our sample’s reduced size, we regard this as a reasonable strategy. Figure 4 shows the network.

**Table 2:** Most frequent keywords.

Keyword	Frequency
Work–family conflict (wfc)	46
Work engagement	18
Job demands	18
Work interference with family life (wif)	18
Gender	17
Burnout	15
Job stress	13
Health professionals	12
Health	10
Workaholism	9
Job resources	8
Mental health	8
Job demands-resources (jd-r) theory	8
Turnover intent	8
Covid-19	7
Well-being	7
Dual-career couples	6
Spillover-crossover effects	6
Work-family enrichment (wfe)	6

Source: Own elaboration.

**Figure 5:**
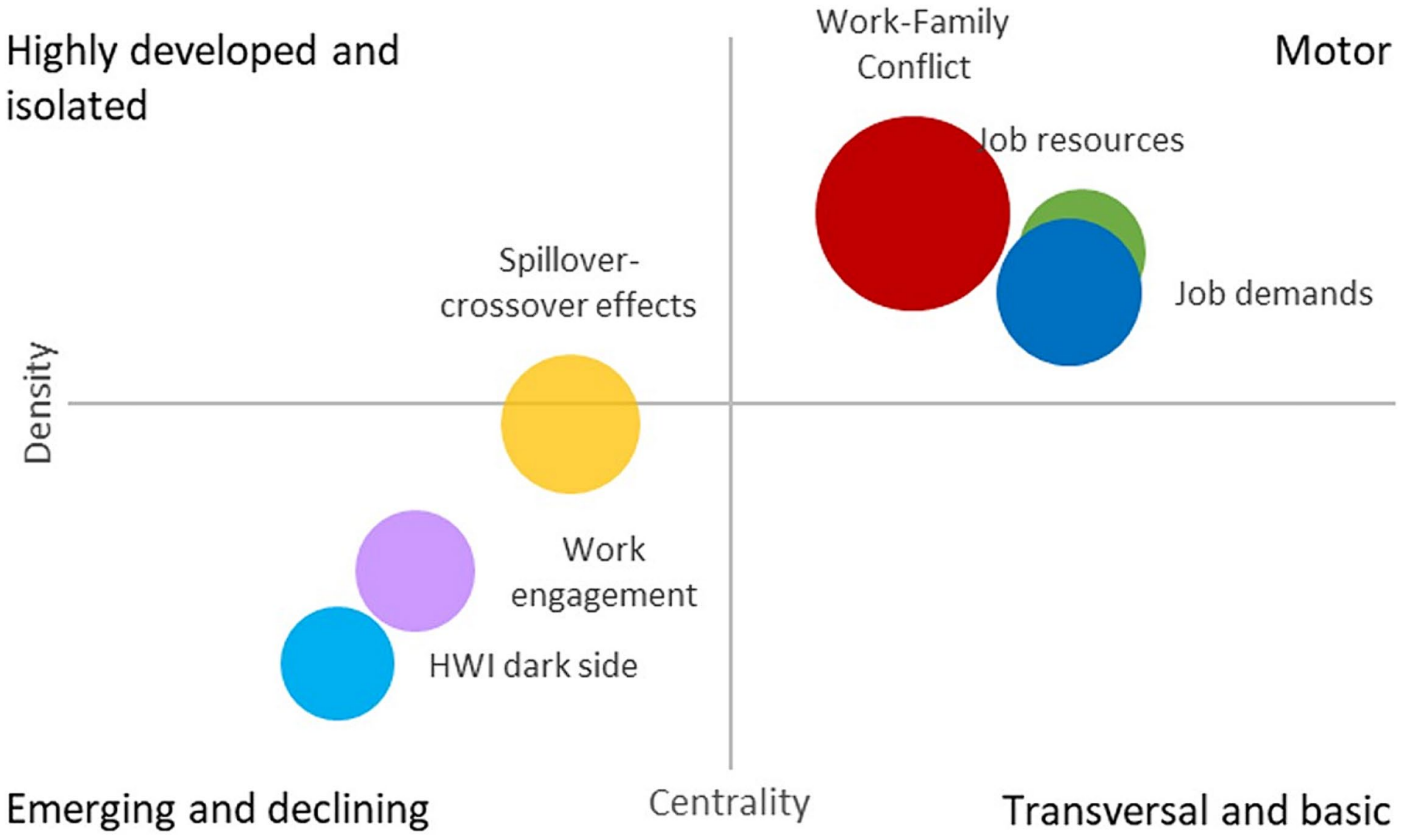
The strategic diagram.

**Figure 6:**
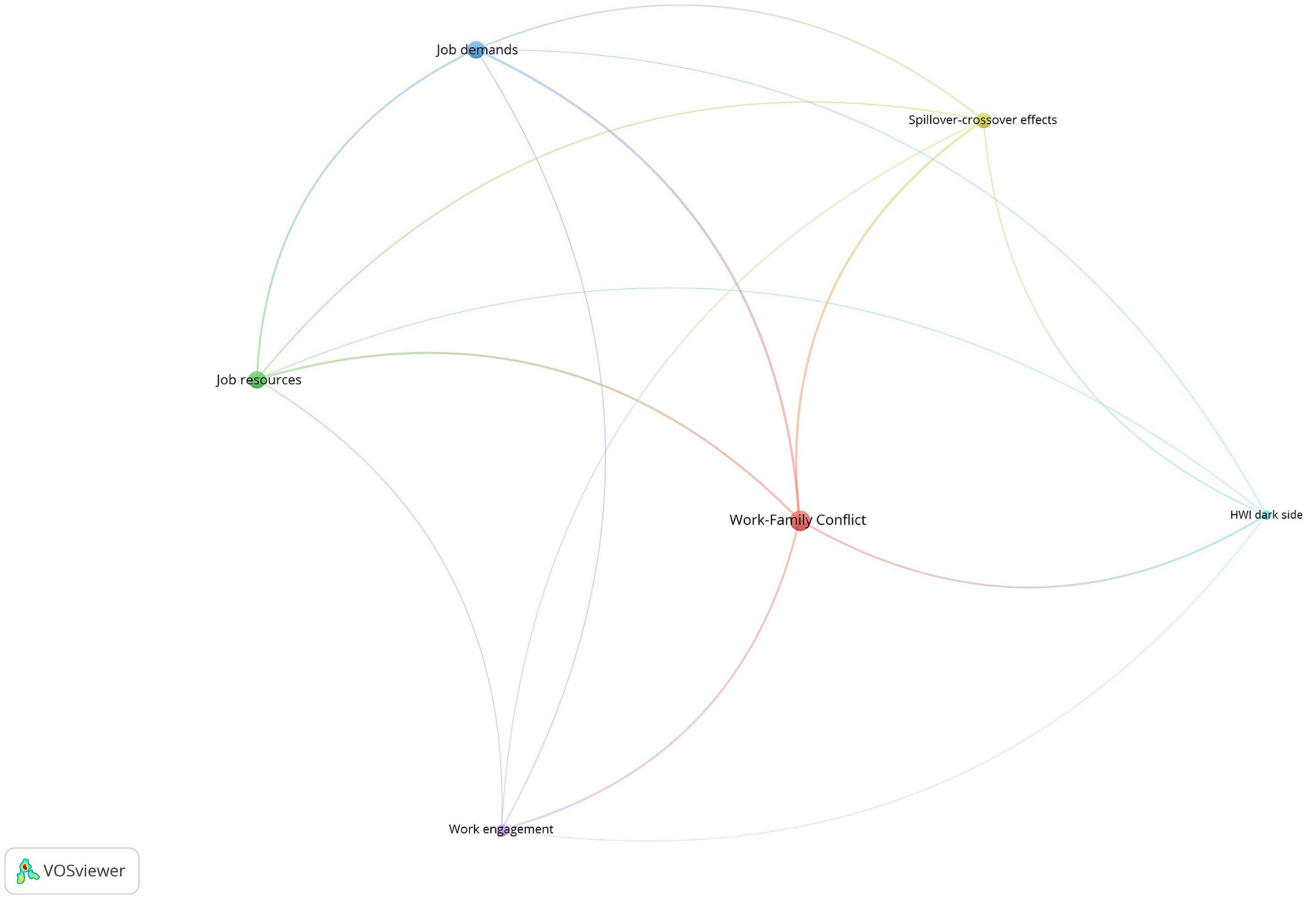
Shrunk network.

The nodes’ color designs the keywords belonging to a cluster. The thickness of the lines is proportional to the strength of the relationship between nodes. The node’s size represents the node’s centrality for the complete network. The VOS algorithm found six different thematic groups. Table 3 details the clusters, and Figure 5 represents the strategic diagram. Finally, Figure 6 contains the shrunk co-word network, representing the relationship between the clusters.

**Table 3:** Relevant information about thematic groups.

Color	Cluster name	Keywords	Articles	Average publ. Year	% after 2021	Average citations per year	% citations after 2021	H index
Red	Work–Family Conflict	17	65	2017.48	50.8%	3.21	43.3%	22
Green	Job resources	13	28	2018.14	50.0%	5.40	50.1%	16
Blue	Job demands	12	37	2013.59	32.4%	3.73	33.7%	21
Yellow	Spillover-crossover effects	10	34	2015.76	41.2%	5.02	39.5%	17
Purple	Work engagement	6	25	2017.75	52.0%	1.82	38.5%	12
Light blue	HWI dark side	4	22	2018.43	54.5%	2.43	39.1%	12

Source: Own elaboration.

### 3.3 Links between areas and clusters

To conduct this study, the keywords presented in 208 articles were analyzed, with the cut-off date being February 2024. Out of this set of articles, the study only works with 83, which constitute the final database. From the preliminary analysis, more than 270 different keywords were obtained, of which 62 were finally considered. The reduction was caused by two factors: substitution of terms with similar meanings and elimination of those with little representativeness because of their low presence or weak relation to HWI.

The relevance of each cluster is given not only by the number of times a keyword is repeated in the selected articles but also by the connection between terms, which allows the creation of networks. This caused the 62 analyzed keywords to be grouped, resulting in six significant clusters. Their size, that is, the number of keywords they contain, is not homogeneous; it decreases, covering fewer keywords as we move forward in the numbering of the cluster.

Of all the keywords analyzed, “Work–Family Conflict (WFC)” is considered the central theme of studies, as the most repeated one in the articles (176 times), followed, albeit by a certain distance, by “Job Demand” (73), “Work Engagement” (70), “Gender,” and “Work Interference with Family Life” (WIF) (both with 69).

The names and characteristics of each cluster have been defined based on the meaning of the keywords they contain, resulting in:

- Cluster 1: Work–Family Conflict and the focus on its adverse effects.
- Cluster 2: The emphasis on Job Resources and their implications on Work-Family Enrichment and Job Satisfaction.
- Cluster 3: The complementary perspective to the previous one. Job Demands and their effects on Job Stress, Well-being, and how Leader Support can mitigate the burden of these demands.
- Cluster 4: The focus on Spillover-Crossover Effects, role transfer between both areas (work/family), and WIF from the dynamics of couples/parenthood and Marital Satisfaction.
- Cluster 5: Work Engagement.
- Cluster 6: The dark side of HWI. Burnout and Workaholism.

The fact that these clusters are organized around key themes such as Work–Family Conflict, Job Demands, and Job Resources largely reflects what was observed in Table 1 with the most cited works. These works could be considered as precursors or seminal in these themes.

We now proceed to conduct a detailed analysis of each one, starting with a reference to their key metrics (summarized in Table 3), particularly focusing on their potential for future development as a research area (refer to Figure 5). Subsequently, the conclusions of the main papers within each group are presented, emphasizing the unresolved issues that will inform future research directions (refer to Section 4.1.2 and Table 4).

**Table 4:** Future research avenues.

	Research questions
Cluster 1: Work–family conflict	Which antecedents and consequences are more relevant for working mothers of infants to experience positive or negative WIF? How does time spent commuting to and from work influence the negative effects of WFC? How does organizational factors such as family-friendly policies and practices influence workers’ experience of WFC and its negative effects? What are the differential effects that age causes on the resilience to stress, burnout, dissatisfaction, and poorer quality of care of health professionals? How do mental health and WFC interact with parenting skills and relationship satisfaction? How do parent and child factors interact over time during the child-rearing period to understand when families are most likely to face mental health issues, burnout, or WFC?
Cluster 2: Job resources	Would cross-domain compensation have the same protecting effect on burnout for men as for women? How do different sources and types of support affect WIF and influence role strain? Could family engagement serve as a link between resources and work-family enrichment? How do different types of leadership and organizational culture impact self-efficacy for home-based remote work?
Cluster 3: Job demands	How do longer work hours impact on work quality? Could downturns in productivity over time be explained by decreases in attention, loss of motivation, greater boredom, or passive aggressiveness toward supervisors? Which psychosocial variables could reduce the impact of work stressors on WIF, thereby potentially facilitating the process of recovery after work? Can control extend to family sphere (e.g., in the form of flexible gender roles, good and affordable childcare or a network of friends and relatives) and change the relationship between job demands and WFC? How do institutional differences between countries affect the level, the nature, and the consequences of job control? How do additional indicators of home demands, as domestic work hours and perceived domestic workload, may influence family satisfaction and psychological distress?
Cluster 4: Spillover-crossover effects	How do spillover-crossover effects change when intergender and intragender couples are considered? Do the effects of a positive attitude toward parenthood and a flexible work situation on general wellbeing and work engagement among working parents differ between different occupational groups and positions? What differential effect do family-responsive policies have on successful management of WIF compared to direct intervention on work-related characteristics?
Cluster 5: Work engagement	How do the combination of individual, group and organizational indicators affect to the relationship between flexible work policies and work engagement? Could men be benefiting disproportionately from on-site work compared to women, given that men are more likely to be “in the know,” receive the mentorship and sponsorship they need, and have their accomplishments noticed and rewarded? How do family members experience women’s work and how does this perception affect opportunities to manage family and work engagement? How do other individual difference variables, such as general self-efficacy and optimism, potentially influence the relationship between family and work engagement?
Cluster 6: The dark side of HWI	How does the presence of children impact the relationship between workaholism and personal burnout over time? Are job flexibility characteristics more valuable for the employee, or might the timing of this job flexibility be better? How might a stronger support system, particularly available to workaholic women with more children, aid in reducing workaholism-related personal burnout?

Source: Own elaboration.

#### 3.3.1 Cluster 1 analysis: work–family conflict

It is the largest cluster in the network, with 17 keywords in 65 out of 83 articles in our database. This group contains the most central node in the network, and part of its node occupies a central position in it (see Figure 4). The average publication year falls in the middle when compared to the other clusters. The same holds true for the rest of the metrics, except for the H-index, which is influenced by group size. The strategic diagram shows its nature as a motor theme, although it is the less central and the densest of the groups in this quadrant (see Figure 5). Its central position in the shrunk network (see Figure 6) and its strong link with the rest of the cluster points to the strong influence of this group in the development of this research domain. According to its metrics, this group has a high potential for the future.

Ten of the 17 words that form this conglomerate are the most important in this study as suggested by the number of times they appear in the articles database (see Table 2). Among them, “Work–Family Conflict” (WFC) stands out, as it is the most repeated, not only in this group but also in the sample of papers. There is no doubt that “Work–Family Conflict” is a fundamental concept within the studies of HWI (as one of its negative outcomes), hence the name of this grouping. Cluster 1 also includes, with a high number of repetitions, other words like “Gender,” “Health Professionals,” “Turnover Intent,” “Health,” and “Emotional Exhaustion” among others, which are linked to mental health (Depression and Anxiety). The concept of WFC is strongly related to gender studies, and papers conclude that factors that influence WFC result in different outcomes for men and women, with no generalizable conclusions applicable across genders (as discussed in lines 171-180). Many articles in the research focus solely on samples comprised of women to demonstrate or confirm the significant impact of WFC on them. Particularly in demanding professions like healthcare, where work-life balance strategies are not always available, employees face heightened pressure leading to less time for rest, leisure, or family. This results in detachment from work, mental and physical exhaustion, and potentially depression. Consequently, workers in such settings often require adjustments to their work-life balance.

WFC literature has paid particular attention to one of the most studied professional groups concerning HWI issues: healthcare professionals (physicians and nurses) (Estryn-Behar et al., 2011; Minamizono et al., 2019; Zhang et al., 2020; Pien et al., 2021). Recent evidence highlights the potential correlation between physicians’ long working hours and the occurrence of significant medical errors and lapses in attention. Considering this, there is a pressing need for future research to delve into the intersection of family and work stressors experienced by doctors. This research should aim to uncover the effects of these stressors on specific health-related outcomes and illnesses among physicians (Milner et al., 2017). Additionally, there is evidence that different practice settings (e.g., health professionals working in educational, or research establishments compared to others) predict the various components of physician burnout (emotional exhaustion, depersonalization, and personal accomplishment) differently (Ádám et al., 2008). Further research into the significance of these differences would be desirable. Regarding nurses, future studies on nurse retention should consider including male nurses, something that is currently anecdotal in research despite their increasing number. This will help to clarify if they experience more psychological stress working in a woman-dominated job (Minamizono et al., 2019).

Additionally, the connection between WFC and other particularly demanding professions is also evident in this group, such as in the case of teachers (e.g., Gu et al., 2020; Saleem et al., 2022; Ratnaningsih et al., 2023; Tang et al., 2023). Rajendran et al. (2020) demonstrate that WFC is the strongest predictor of emotional exhaustion for male and female teachers. In the case of Cinamon and Rich (2010), authors reaffirm the impact of WFC on teachers’ emotional health, emphasizing the role of managerial support in mitigating this impact.

It is important to underline the connection of the topic WFC with those terms found in Cluster 2 (Job Resources) and Cluster 3 (Job Demands). It’s worth noting that the Job Demands-Resources model is essential in this literature for understanding WFC.

#### 3.3.2 Cluster 2 analysis: job resources

It is the second-largest cluster in the network, with 13 keywords. The number of articles that deal with these topics is far less than the previous cluster. However, the average year of publication renders it as one of the youngest groups. Its position in the strategic diagram and its metrics confirm this group’s high potential for development and the central role that it has already played in the field (see Figure 5). The analysis of the shrunk network points out that the most substantial relationships of this group are to the WFC cluster, and the Job demands one (see Figure 6).

Among the 13 keywords gathered in this cluster, the importance of “Job Resources” stands out as the most repeated (38), closely followed by “Job Demands Resources Theory” (37) (two of the most frequent keywords in Table 2). This model constitutes the fundamental theoretical framework in research on HWI, with its three parts: antecedents, dimensions, and outcomes (Acosta-prado et al., 2021). Concerning the former, “Conservation of Resources Theory” (27) stands out since it underscores the emphasis on job resources and their implications in the enrichment of work-family relationships and job satisfaction. This cluster focuses on the positive outcomes of HWI and some antecedents that may enhance work-family enrichment (as including cross-domain compensation, support, remote work, autonomy, and flexibility).

Different studies in this field highlight the integration of factors not currently considered within the theoretical models of enrichment to enhance research. For instance, Lapierre et al. (2018) call for expanding research on role salience (work/family centrality) and its influence on relationships between broader (macro) contextual characteristics (e.g., societal culture, a country’s level of economic prosperity, governmental policies), personal (micro) characteristics (e.g., self-efficacy, social power, optimism, occupational status, social class), and WFE. Likewise, Dishon-Berkovits (2014) propose advancing our understanding of how cross-domain compensation (as a part of work-family enrichment) can contribute to healthier outcomes within the work domain by reducing job burnout and promoting individual as well as organizational well-being. Gordon et al. (2012) propose that all sources and types of support (e.g., emotional support from supervisors, coworkers and unpaid instrumental support from family and friends, emotional caregiving support from family and friends, paid instrumental support, and instrumental support from the workplace) must be simultaneously considered to better understand the cross-domain impact of both work and support. The dominance of the JD-R framework in research within this field shapes the factors under analysis. Therefore, delving more deeply into alternative perspectives such as the Conservation of Resources theory, Role Conflict theory or Resource Drain theory could enrich research in this area.

Another important term in this group is “COVID-19,” present as a keyword in 30 articles. This conglomerate groups naturally terms related to remote working, promoting autonomy, job satisfaction, and literature focus on the changes in work-life balance and family relationships (Syrek et al., 2022; Vitória et al., 2022). In the case of Tsang et al. (2023), the focal point lies in the effect of remote work on the family-work relationship, impacting self-efficacy and productivity, along with the recognition of influencing factors and mediators in these relationships. This study underscores the importance of supervising remote work hours and self-regulation to enhance work-from-home productivity, besides confirming gender differences. As we will see in the Discussion section, much of the future potential of this topic is associated with the unanswered questions regarding the effects of remote work on job demands and job resources.

#### 3.3.3 Cluster 3 analysis: job demands

This group occupies a less central position in the network (see Figure 6). Although its centrality and density characterize it as a motor theme (see Figure 5), its metrics show that its potential for future development is moderated, with fewer documents published in the last three years and a decreasing number of citations. It is the oldest group, with an average publication year of 2013.59 (see Table 3). The closest groups to this cluster are the Work–family conflict and the Job Resources one.

This cluster focuses on various negative outcomes of HWI and different antecedents that may reduce stress and improve employee well-being (such as leader support and control mechanisms). It gathers keywords mainly related to “Work.” “Job Demands,” “Job Stress,” “Well Being” and “Leader Support” clearly stand out, as well as the relationship among three of them: demanding jobs with high demands can cause job stress for responsible workers. Within the different job categories, team leaders or department leaders are among the roles most affected in all organizations because of their level of responsibility. In such instances, providing support for leaders is crucial, as they typically face greater pressure within organizations. Conversely, there is an inverse relationship between demand and responsibility and workers’ well-being.

Another keyword is “Job Control”: establishing job control mechanisms, and incorporating work-life balance measures, would allow for a reduction in stress and an improvement in employee well-being. As expected, both “Job Demand” and “Job Stress” are present in papers also linked to Clusters 1 (WFC) and 2 (Job Resources). On the one hand, this relationship is logical since a demanding job, with responsibility in the company, means that workers may have less time both for rest and for family attention, which would cause WFC. On the other, being stressed can generate anxiety and even depression, which logically leads to a decrease in well-being. Thus, several models of job stress and strain highlight social support as a key resource to assist employees in managing job strain (Roxburgh, 1996). Many authors work with the Job Demands-Resources (JD-R) Theory.

Work hours have garnered a lot of attention from researchers as a key indicator of job demands. Therefore, it is around this concept that prospective opportunities for improvement in research arise. It would be very useful for future researchers to explore the effects of longer work hours on work quality, including potential decreases in attention, motivation, increased boredom, or passive aggressiveness toward supervisors (Ng and Feldman, 2008). Prior research found weak links between work hours and well-being. Social support and other protective factors might also influence the relationship between job demands and WIF (Hughes and Parkes, 2007). Further research is needed to identify psychosocial variables that lessen the impact of work stressors on WIF, potentially facilitating the process of recovery after work.

The studies in this field have significantly incorporated the role of control in the relationship between job demands and their consequences, such as well-being, attention to family demands, and stress. However, it is not just about control within the tasks but also about control over how the work is organized and how many people are on the team, especially in flexible and unclear job roles (Grönlund, 2007a). Besides, with organizations quickly changing these days, it is important to study what happens when people feel they have less control over their tasks and performance. Another important topic for further research is whether work allows flexibility for family needs, such as schedule and workload adjustments or to care for a sick child. This kind of measures would need to come with a corresponding control in the private sphere, like flexible gender roles, affordable childcare, or support from friends and family, which can help in sharing caregiving responsibilities when needed (Grönlund, 2007a). Future research should also explore the possibility that institutional differences between countries could affect the level, the nature, and the consequences of job control (Grönlund, 2007b).

#### 3.3.4 Cluster 4 analysis: spillover-crossover effects

This cluster is the closest to the strategic diagram’s center, which makes it difficult to classify (see Figure 5). Despite the fact that it is in the emerging and declining themes quadrant, it has many documents and citations (see Table 3). However, the proportion of recent documents and citations is smaller than most motor themes. All these figures characterize it as a mature topic with a limited potential for future development, although its academic resonance is among the highest. This cluster has the most solid relationships with the previous groups, and it does not have a connection with the HWI dark side cluster.

This cluster gathers the crossover effects between work and family, as the latter is understood as a relationship with the partner and/or in the care of children. It is focused on the transfer mechanisms between work and family domains, with an emphasis on role distribution within couples and their effects on each other. The most significant keywords are “Work Interference with Family Life” (WIF), “Dual-Career Couples,” and logically “Spillover-Crossover Effects” (all of them included as most frequent keywords in Table 2). Research in this field indicates that many employers offer family-friendly policies like maternity and parental leave, childcare programs, flexible schedules, and support services, which are appropriate for dealing with family demands and role work distribution, and consequently for reducing the negative influence of family life on work. While these practices can assist employees in balancing both aspects of their lives, organizations should also consider work-related factors (job demands and job resources) that may contribute to conflicts between work and family life (Eek and Axmon, 2013).

Also prominent are “Marital Satisfaction,” “Parenthood,” and “Stress,” which are terms that emphasize the meaning of the group and are related to the distribution of roles. Ratnaningsih et al. (2023) investigate the spillover-crossover effects on the WIF, with an emphasis on WFC and FWC on marital satisfaction and personal burnout. Their results show that there was no spillover-crossover effect of WFC and FWC on marital satisfaction for both working wives and husbands. Meanwhile, Bakker et al. (2009) investigate the existence of direct crossover effects of relationship satisfaction between partners, confirming it positively. Regarding the link between parenthood and stress, Eek and Axmon (2013) identify that workplace factors related to flexibility and, particularly among women, attitudes toward parenthood seem to have the most significant effect on working parents’ subjective stress and well-being, while benefits appear to have a lesser impact.

From this perspective, cultural, institutional, and social influences may also play an important part, particularly when addressing societies with very traditional norms and a clear division of roles (Choi, 2008; Ratnaningsih et al., 2023).

#### 3.3.5 Cluster 5 analysis: work engagement

This cluster is placed in the emerging and declining quadrant of the strategic diagram (see Figure 5), and its metrics show its emerging nature. It is one of the youngest groups, and that explains its smaller number of citations (see Table 3). Besides, this group is less well-defined than the previous ones, and the prevalence of the term “work engagement” dominates in it. Its position in the network, the distribution of its nodes, and its place in the shrunk network show this double nature with a very central element and some satellites that have had less importance in the field.

This group was initially formed by six keywords. For interpretation purposes, one of them stands out for its significance, “Work Engagement,” giving the name to the group (the second most frequent keyword as Table 2 shows). Additionally, “Flexible Work” and “Family Centrality” are prominent, although to a lesser extent. This cluster focuses on this dimension of HWI and on several individual and organizational antecedents that may contribute to engagement, such as family centrality and flexible work. The fact that workers exhibit a strong sense of belonging to the company or organization where they work may be related to the work-life balance measures it provides. The need for talent retention by companies means that they favor and support flexible work, which in turn is related to family commitment. The work flexibility measures implemented facilitate a balance between work and family.

The fact that the dominant perspective in the association between work engagement and flexible work revolves around executive women (their experiences and behaviors) only emphasizes the importance of delving deeper into the study of gender differences in this relationship. This calls for an expansion of studies that incorporate a systemic and multilevel perspective of the work environment (individual, group, organization) and include dyadic studies in the family or private domain (Lu et al., 2011; Dåderman and Basinska, 2016; Costantini et al., 2021). In this line, there is an interest in investigating possible detrimental effects for women of extension of flexible work arrangements, in comparison to men working on-site. According to Field et al. (2023), men could be benefiting disproportionately from on-site work compared to women, because males are more likely to be “in the know,” receive the mentorship and sponsorship they need, and have their accomplishments noticed and rewarded when they work on-site.

#### 3.3.6 Cluster 6 analysis: the dark side of HWI: burnout and workaholism

It is both the youngest cluster in the network and the smallest one, with the smallest number of articles and keywords. As in the previous case, we can see a division between the nodes, with two topics, “burnout” and “workaholism,” very central and connected with the rest of the network (except for the Spillover-crossover effects cluster) (see Figure 4). That characteristic and its metrics point to its transversal nature, although it is in the emerging and declining theme quadrant. The association between both concepts is consistent with the evidence from previous literature (e.g., Galdino et al., 2021).

The two keywords with the most weight in this cluster are “Burnout” and “Workaholism,” important terms in the studies of HWI (see their relevance in Table 2). The connection of both concepts with others in Cluster 1 is clear. As mentioned earlier, Cluster 1 includes both physical and mental exhaustion, as well as mental health-related aspects such as anxiety and depression. There is evidence that WFC can cause or intensify both effects. Regarding psychosocial correlates, workaholism is positively associated with work–family conflicts (Russo and Waters, 2006; Andreassen et al., 2013; Chang et al., 2023). According to Eason et al. (2022) both work addiction and burnout have potential negative consequences on personal health. In most cases, the analysis includes a study by gender, which highlights stronger repercussions in women. It also affects more significantly among professionals in the care and health sector, as a result of the type of tasks to perform, dedication, and involvement. Accordingly, a high number of papers in this group are related to health professionals, because of their special exposition to these aspects.

As for the link with terms in Cluster 4, on one hand, the relationship with Burnout is supported by job exhaustion and stress it generates, which interrelates with elements specific to Cluster 4 (such as WIF, working couples, and parenthood). On the other hand, Robinson et al. (2006) in their study on the experience of women married to workaholics, concluded that the strength and cohesion of a marriage were associated with the presence or absence of workaholism, and that the intersection between workaholism and marriages was an area of empirical and clinical research that was still largely overlooked.

The central paper of this group is Eason et al. (2022), which links work addiction with WFC and burnout. With a specific focus on athletic training, this study concludes that women were more at risk for compulsive tendencies than men. Other studies also simultaneously address both effects. (e.g., Russo et al., 2023). Alongside this type of research, others within this group focus either on workaholism (Bakker et al., 2009; Shimazu et al., 2011; Huml et al., 2020; Xu and Li, 2021; Falco et al., 2022) or on burnout (Ádám et al., 2008; Houkes et al., 2008; Dishon-Berkovits, 2014; Nilsen et al., 2016; Beauregard et al., 2018; Minamizono et al., 2019; Abusanad et al., 2021; Lee et al., 2021; De Beer et al., 2023).

## 4 Discussion and future research avenues

In this review, we have identified the key characteristics of the literature at the intersection of HWI, gender, and flexibility, considering the evolution of production and paper impact, authors and their origins, journals, main cited articles (intellectual foundation), and topics addressed. With a variable production over time and great diversity in terms of journals [similar in variability to other literature reviews, such as those by Kişi (2023) or Nguyen et al. (2023)], the fundamental relevance of works in this field may have never been higher than at the beginning of the 21st century, a phenomenon that is also confirmed in other literature reviews (Reimann and Diewald, 2022; Gonçalves et al., 2023).

The identification of the most cited authors aligns with the most central and prolific themes in the reviewed literature. Eighty-six percent of these works are related either to the Job Demands-Resources model or to WFC. The exception to this predominance is represented by two methodological studies on measurement tools for work engagement and burnout (Schaufeli et al., 2002, 2006), which prominent use is justified by their applied nature, and the seminal work on burnout by Maslach et al. (2001) which provides a critical review of 25 years of research on job burnout.

In recent decades, significant changes in family and work dynamics have occurred across societies. These variations include shifts in family structures (more diverse family types and the evolving role of fathers), as well as transformations in the labor market, including increased female workforce participation and greater work flexibility. Additionally, welfare states have responded with policies aimed at promoting gender equality and supporting work-life balance, such as expanded childcare services and flexible work arrangements. However, work-family balance still implies tensions for individuals, especially when other family members are involved (Reimann et al., 2022). This fact supports the ongoing attraction of researchers toward the domain of WFC. Despite its roots in the mid-20th century, we conclude that WFC remains the most central and prolific area, with potential for future development.

It is worth noting the shift in interest regarding the analysis of the two parts of the Job Demands-Resources model. Notwithstanding the apparent decline in interest in the former, the focus in the analyzed literature clearly shifts toward the side of Job Resources, showing obvious potential for the future. We conclude that in the context of talent scarcity and efforts to retain employees, priority is being given to better understanding the individual and organizational factors that can facilitate work-life balance. For instance, in the specific case of female STEM talent, Singh et al. (2018) conclude that, in response to the talent shortage, corporate leaders and lawmakers are advocating for heightened initiatives to attract and retain women engineers. They assert the need to thoroughly assess organizational factors that may impact their occupational attachment and withdrawal decisions.

We surmise that the growing importance of organizational support in improving work engagement is unquestionable, with initiatives such as autonomy, career development encouragement, and acknowledgment of diversity. Luo et al. (2024) emphasize the importance of institutional structure and system support in fostering employee engagement. Furthermore, Tsubono et al. (2024) underscores the role of organizational job resources in shaping workplace social capital, with organizational-level resources being particularly influential in driving work engagement. Additionally, Chauhan et al. (2022) underline the need to understand how both family and organizational factors can impact women’s career progression. The importance of organizational support programs, career planning, and growth activities in facilitating career success for women is also noted. However, limited attention has been paid to gender differences in organizational support for work-family needs (Clark et al., 2017), so more research is crucial to encourage organizations to adopt a robust family-friendly culture and implement formal family-friendly policies.

The explicit consideration of a gender perspective in literature entails nuanced differences regarding the geographical origin of the works. We conclude that the emergent prominence of studies on China and Japan when the focus of the review is more specific on gender is clear, although studies in this field predominantly come from Western countries (particularly North America and Europe), similarly as with the general literature on HWI (Choi, 2008; Cohen et al., 2023; Mori et al., 2024). In China, women often prioritize their family roles above work ones. With increasing competition in the professional arena, women may face heightened vulnerability to WFC compared to men, a context that increases interest in analyzing women’s experience in China (Zhang et al., 2019; Lin et al., 2020). Contemporary Japanese society shows a corporate-centric and male-dominant structure with significant gender role division in labor. Nevertheless, women’s increasing economic independence and social participation do not alter existing gender roles but instead reinforce them, as they support the corporate-centered society through employment while shouldering the majority of domestic labor, perpetuating male dominance (Ogiwara et al., 2008; Nakamura et al., 2022). These cultural, social and institutional singularities have also led to numerous studies aimed at validating measurement scales for antecedents, dimensions, and consequences of HWI in these countries, typically designed for Western nations, further increasing the visibility of research output in Asian countries (Andersen et al., 2023).

We emphasize the importance of remote work options and flexible work arrangements for women and consider them strategic in promoting women’s careers. The strong link between work engagement, flexible work, and women executives shown in Cluster 5 is consistent with the findings of the latest McKinsey report, “Women in the Workplace 2023” (Field et al., 2023). According to this study, many employees prioritize remote work opportunities and schedule control as significant company benefits, second only to healthcare. Women, in particular, value these benefits more, likely because of their disproportionate responsibility for childcare and household tasks. Workplace flexibility is crucial for many mothers that, without it, would have to leave their jobs or reduce their hours. However, it is not just women or mothers who benefit; hybrid and remote work offer valuable advantages to most employees. Both women and men highlight better work-life balance as a primary benefit, along with reduced fatigue and burnout. Research indicates that achieving good work-life balance and minimizing burnout are essential for organizational success.

Finally, in the evolution of the topics addressed in this research field, there is a turning point in the year 2020. The COVID-19, insofar as it has significantly altered the dynamics of the work-family relationship and the hours dedicated to each sphere, hand in hand with remote work alternatives, flexible work arrangements and limiting socialization and support options, has attracted much of the attention of the most recent academic discourse. The interest in the different consequences that these factors have for women and men are behind the significant recent boost in this literature.

### 4.1 Future research lines

Based on our review of the HWI-gender-flexibility research, we also propose new questions that may lead to new research opportunities, summarized in Table 4. Detailed discussions on our primary methodological and theoretical recommendations for future studies follow below, starting with some overarching issues and then proceeding to the specifics of each domain.

#### 4.1.1 Methodological and cross-cultural opportunities

The widespread use of cross-sectional studies implies that the results represent only one point in time, overlooking the impact of time on the relationship between variables. Particularly, measuring variables at several points in time could facilitate the identification of causal relationships and their reciprocal direction. Future research based on longitudinal lens, intervention studies or multiwave designs would be particularly useful in shedding light on the causal sequencing of variables included in these studies (Eek and Axmon, 2013; Lapierre et al., 2018; Huml et al., 2020; Eason et al., 2022). Additionally, the predominance of questionnaire-based studies that essentially collect measures of individuals’ perceptions reflects a gap in qualitative studies that would be desirable to explore in future research. As studies are generally based on self-reporting (subjective data) of behaviors and performance, this creates concern for method bias. Future research would be strengthened by inclusion of objective outcome measures to reduce the methodological limitations of self-report data (Hughes and Parkes, 2007; Hakanen et al., 2011; Lu et al., 2011; Halliday et al., 2018; Saleem et al., 2022).

Different cultural and workplace factors can influence people’s perceptions. For example, differences in individualism/collectivism in work groups, or high/low gender egalitarian countries have proven to be relevant in explaining the different relationship between variables such as job autonomy and job outcomes (Man and Lam, 2003; Au and Cheung, 2004; Halliday et al., 2018). According to Bakker et al. (2008), social, cultural, and political contexts may affect individuals’ perceptions and experiences within the work–family domain. In the future, researchers should investigate which specific factors in different cultures are most important for how women and men experience HWI and its consequences.

#### 4.1.2 Thematic developments

This section is devoted to present unresolved issues identified in our review of the HWI-gender-flexibility research related to each thematic specific domain (each cluster). These areas represent potential opportunities for future research and are formulated as questions in Table 4.

##### 4.1.2.1 Work–family conflict

The imbalance between studies about positive versus negative interaction between work and family is pronounced, with a predominance of research conducted from a conflict perspective. According to Hakanen et al. (2011), there is a clear necessity for longitudinal studies that comprehensively explore both the positive and negative bi-directional interactions between work and family. In this literature, research on working mothers of infants is generally missing, which, according to Carlson et al. (2011), represents an opportunity, as this group provides a good representation for testing models of the work-family interface. The transition in family structure and interaction serves as a natural experiment to examine potential antecedents and consequences of work-family experiences.

For other authors, future research on work–family conflict should explore in depth the personal demands in WFC, given findings that it was the strongest predictor of emotional exhaustion for both men and women (Rajendran et al., 2020). Stress perceived from demands within and outside the job domain should be investigated alongside each other, as the different stressors do not disappear when one leaves work or home, potentially exacerbating stressors within the other domain. For instance, time spent commuting to and from work can influence job stress. With the same purpose of delving into the relationships between factors that influence workers’ experience of WFC and its negative effects, Pien et al. (2021) propose advancing the examination of organizational factors such as family-friendly policies and practices. Considering not only active groups of workers but also those who have already resigned would significantly advance our understanding of turnover intent, including their reasons for leaving.

As to the potential research about professions with higher risk of negative outcomes of HWI, concerning nurses, it would be interesting to further investigate the effect of age on their resilience to stress, burnout, dissatisfaction, and poorer quality of care. Studies on student nurses have indicated that they are more affected by workplace violence experiences and report more distress compared to older nurses. Therefore, future studies should incorporate psychosocial factors and violence as significant withdrawal drivers.

If the focus is on health (physical and mental) because of WFC, the literature raises some questions for which there is still insufficient evidence. For instance, future research should investigate how mental health and work–family conflict interact with important factors related to partners and children, such as parenting skills and relationship satisfaction. This knowledge can be valuable for informing policy making and prevention efforts before cross-over effects to other family members can take place (Nilsen et al., 2016). In addition, further research is needed to look at how parent and child factors interact over time during the child-rearing period to understand when families are most likely to face mental health issues, burnout, or WFC.

Finally, the focus on gender rather than sex in these studies could enhance the comprehension of masculine and feminine behavioral patterns. Sex serves as a proxy variable for the broader conceptual construct of gender, but it is essential to recognize that women can exhibit masculine traits, just as some men can display feminine characteristics. This shift in perspective allows for a more nuanced exploration of gender dynamics and their impact on behavior (Houkes et al., 2008).

##### 4.1.2.2 Job resources

From the perspective of how job resources can facilitate WFE and job satisfaction, an avenue for future research would be to delve into the role of certain moderating factors. For example, family engagement could link the relationship between resources and work-family enrichment (Siu et al., 2010), or different types of leadership and organizational culture could impact on self-efficacy for home-based remote work (Lange and Kayser, 2022), broadening the perspective on additional aspects that promote personal resources of employees.

Additionally, given that the COVID-19 pandemic has significantly transformed job resources and job demands, research on the effects of remote work has gained prominence, and suggestions for future development are proposed. Specifically, regarding the impact on job-related stress, well-being, and quality of life, Orfei et al. (2022) suggest studying the effect of possible sleep disorders caused by changes in daily schedules related to the reorganization of individual and family routines in homeworking. Additionally, including the perspectives of employers and supervisors in data collection concerning practices for remote work is deemed urgent, as emphasized by Vitória et al. (2022). There is a call for organizations and managers to recognize their role if they allow employees´ homeworking, recognizing that the work arrangements should be an employee’s decision. The perspective of practitioners supports these suggestions. The “Women in the Workplace 2023” report highlights this divergence between employees and the organization. While 83 percent of employees say the ability to work more efficiently and productively is a primary benefit of working remotely, only half of Human Resources leaders say employee productivity is a primary benefit of working remotely (Field et al., 2023). In order to unlock the full potential of flexibility, this report emphasizes that organizations and managers must: (a) Establish clear expectations and norms around working flexibly; (b) Measure the impact of new initiatives to support flexibility and adjust as needed; and (c) Ensure equal opportunity and fair evaluation systems across work arrangements (avoiding the flexibility stigma and focusing on results, rather than when and where work gets done).

##### 4.1.2.3 Job demands

From a methodological perspective, there are several avenues for future development. In general, more multi-source measurements and multi-item scales are needed to enhance the methodological rigor in this field of research. To better understand modern work conditions, how job demands are measured should be improved. Instead of just looking at time pressure, future research could also consider other factors like the need for learning and adapting, as well as the emotional effort required (Grönlund, 2007a). Additionally, Ng and Feldman (2008) propose that measures for the quality of work hours should be improved because they are not measured relative to the number of work hours. According to these authors, ideal measures of quality of work hours should capture the quality of work output relative to the time input. Likewise, the use of additional and holistic indicators of family demands (e.g., domestic work hours or perceived domestic workload) is also required to advance knowledge about the influence of work hours in family satisfaction and psychological distress (Hughes and Parkes, 2007).

Additionally, future research could explore demand factors that are more job-specific than general. Besides the specific tasks associated with stress for each profession, the investigation would have to address the when and how often those tasks are undertaken, together with an exploration of the environments (Bowen et al., 2014). Even more, different work settings (leadership styles, the kind of organization of work, human resources policies of the company, etc.) and diverse family situations (support from spouse, distribution of domestic tasks, domestic burden, etc.) should be combined in future research to identify different experiences of stress (Chela-Alvarez et al., 2020).

##### 4.1.2.4 Spillover-crossover effects

The focus of studies in this domain on couple dynamics, using both members of the couple as sources of information, creates the first opportunities for future research. Since most studies focus on intergender relationships, the extension of the results to same-gender couples is unknown, calling for an expansion of research in this direction (Bakker et al., 2008, 2009). Likewise, the use of small samples is noted (because of the difficulty to obtain responses from two working parents with busy schedules) as well as the limitation of positions within the organization being analyzed (for example, employees or business owners with no employees, first-line managers, and middle-level managers), offering opportunities for future work with more sophisticated and holistic samples (Eek and Axmon, 2013).

As we discussed in the analysis of Cluster 4, the conclusions of the studies in this line indicated that while the family-friendly policies developed by organizations can mitigate the negative influence of family on work, they also called for considering work-related factors. Further research on the effects of these characteristics (such as workload, uncomfortable work environment, mentoring or coaching, training, job control, regular constructive feedback, and clearer goals) will help support organizational strategies.

##### 4.1.2.5 Work engagement

Regarding methodological issues, studies in this field are generally based on self-reporting (subjective data) of behaviors and performance, and this creates concern for method bias. To remove this limitation, future research can consider the collection of more objective indicators by combining data from both managers/leaders and employees. Additionally, the focus is mostly on the availability of certain measures within the organization, rather than on their actual use. Employees’ awareness and interpretation of the signals sent through the availability of policies (such as flexible work arrangements, for example) can influence both work engagement and organizational commitment (Costantini et al., 2021). Future studies should measure interpersonal communication and everyday actions and employ processual designs to conduct path analysis, thus improving the possibility of informing about the causal nature of the relationships.

##### 4.1.2.6 The dark side of HWI

From the works addressing this topic and exploring the relationship between workaholism and burnout, research opportunities arise on unresolved issues. One of them concerns the predominant methodology applied in these investigations as we mentioned before. Therefore, future examination of the concept of work addiction and burnout from a longitudinal lens would be beneficial. Besides the methodological issues, the conclusions of these studies suggest that organizational, rather than individual-level factors, may be influencing the work-addiction risk (Eason et al., 2022). One factor that remains unexamined in these works is the concept of flexible scheduling, which is referenced as a buffer of work–family conflict. Another potential omission could be the presence of a supportive work environment. The level of support within the work sphere has been recognized as a factor influencing work addiction. Consequently, future studies examining the impact of job flexibility and support as organizational factors that explain women’s risk for compulsive tendencies in comparison to men could be more valuable (Huml et al., 2020; Russo et al., 2023).

## 5 Conclusions and implications

This study advances our understanding of the intersection between HWI, gender, and flexibility literature. Firstly, it confirms that despite changes in family and work dynamics, WFC remains central in the literature, and continues to attract research interest. Secondly, by identifying a shift toward job resources research, it suggests a certain prioritization of facilitating work-life balance amidst talent retention efforts. Thirdly, it highlights the increasing importance of organizational support, including autonomy, career development encouragement, and diversity acknowledgment, in boosting work engagement and women’s career progression. Fourthly, it signals a growing prominence of research addressing gender differences in the intersection of work and family in non-Western countries, especially in those with a distinctly traditional and conservative culture, where roles and responsibilities are clearly defined, and where the dominant burden of family life continues to fall on women. Fifthly, it underscores the importance of remote work options and flexible work arrangements for women and considers them strategic in promoting women’s careers indeed, and not simply facilitating work-life balance features. Nevertheless, it does not ignore the potential downside effect of these actions if they exclude women from the space of evaluation, recognition, and promotion that male can exploit while working in person.

All in all, this work yields significant contributions, both from an academic perspective and for practitioners, organizations, and governments. Academically, we propose interesting lines of research that can build upon existing knowledge. Methodologically, this work calls for adopting new alternatives beyond the dominant cross-sectional studies, which have significant limitations in establishing bidirectional causal relationships due to their static nature. Additionally, there are many options to advance in improving the measurement of variables, mostly relying on self-reports and perceptions. Broadening the scope to include a more comprehensive examination of gender (as a continuum of masculinity and feminism behaviors) and cross-cultural studies will enable us to draw more robust conclusions about the specificity of women and men regarding HWI. Likewise, for each of the domains identified through clusters, new specific questions are proposed for further investigation.

Regarding implications for practitioners, organizations, and governments, the current landscape is characterized by talent competition and efforts to retain employees Improving the understanding of individual as well as organizational elements that contribute to achieving work-life balance must be a strategic priority for organizations and governments. This comprehension is crucial for defining and implementing the most effective measures, while acknowledging gender diversity. Organizational support, including autonomy, encouragement of career development, and recognition of diversity, is increasingly recognized as vital in boosting work engagement. Moreover, the significance of institutional structures and system support in fostering employee engagement cannot be overstated. The role of team leaders and HR managers as key players in this system of employee support is crucial. Moreover, while the identification of measures in place is relevant, the implementation and estimation of their effectiveness is critical. There is a clear call for organizations and institutions to go further with a systematic evaluation of the impact of their initiatives.

## Data availability statement

The raw data supporting the conclusions of this article will be made available by the authors, without undue reservation.

## Author contributions

CE-G: Writing – review & editing, Writing – original draft. LF-R: Writing – original draft, Writing – review & editing. J-JN-S: Writing – original draft, Writing – review & editing.
