# Supplementary material for: Incorporating gendered analysis and flexibility in heavy work investment studies: a systematic literature review
Source: Front Psychol. 2024 Jun 19;15:1401201. doi: 10.3389/fpsyg.2024.1401201 (PMC11220130; doi:10.3389/fpsyg.2024.1401201)
Supplement: Supplementary file 1 [file Table_1.docx]

Supplementary Material

Incorporating Gendered Analysis and Flexibility in Heavy Work Investment Studies: A systematic literature review

Carmen Escudero-Guirado^1*^, Lourdes Fernández-Rodríguez ^1^, Juan-José Nájera-Sánchez^2^

^1^Facultad de Ciencias Económicas y Empresariales, Universidad Pontificia Comillas, Madrid, Spain

^2^Facultad de Ciencias de la Economía y de la Empresa, Universidad Rey Juan Carlos, Madrid, Spain

*** Correspondence:**
Corresponding Author
cescudero@comillas.edu

# Supplementary Figures and Tables

## Supplementary Tables

**Supplementary Table 1.** Articles included in this review in the intersection of HWI, gender and flexibility.

| **Authors** | **Objectives** | **Sample** | **Context** | **Kind of study** | **Unit of analysis** | **Results** | **Discussion and implications** | **Clusters (num. of topics)** |
| --- | --- | --- | --- | --- | --- | --- | --- | --- |
| **Demerouti et al. (2005)** | To research spill over of stress transferring from work to home and crossover research of strains transferring from one spouse to another. | Couples dual-earner parents (N=191) | Netherlands | Quantitative | Family | Structural equation modeling supported the mediating role of WFI although there were genders differences. | Observed results implied not only job-related strain, but also positive, context-free well-being may crossover among partners. | 3(1); 4(4) |
|  |  |  |  |  |  | Crossover path from females’ exhaustion to males’ exhaustion and from males’ life satisfaction to their partners’ life satisfaction. |  |  |
| **Day and Chamberlain (2006)** | To explore direct and indirect relationship of role commitment with work–spouse and work–parent conflict. | Female nurses and police officers | Canada | Quantitative | Individual | Negative effects of job stressors on conflict would be somewhat alleviated by high job and spouse commitment but aggravated by high parent commitment. | Research needs to be conducted to determine whether the relationships found in the present study are applicable to men and to employees in other occupation. | 1(2); 3(1); 5(1) |
| **Pal and Saksvik (2006)** | To study gender and cultural differences experienced in the work-family conflict (WFC). | Norway: Doctors (N=27) Nurses (N= 328). India: Doctors (N=111) Nurses (N= 136) | Norway and India | Quantitative | Individual | Norwegian nurses experienced high job demands associated with WFC and Indian nurses perceived high job control and low flexibility in working hours associated with WFC. | No gender difference visible between the doctors and nurses of both countries into the perception of WFC. Differences in the job control and flexibility in working hours on the work-family conflict between geographies. | 1(2); 3(2); 5(1) |
| **Grönlund (2007a)** | To analyse effect of job control on working hours, work-family conflict and psychological wellbeing. | Swedish employees (N=800) | Sweden | Quantitative | Individual | High job demands are associated with longer work hours, more work-family conflict and lower wellbeing, while control has positive effects, even when demands are high. | Interaction variables confirm that gender differences are significant at the two middle levels of control. | 1(1); 3(4); 5(1) |
| **Grönlund (2007b)** | To analyse how not only the quantity of work but also its quality is key, as captured by the job demand–control model. | Swedish employees living on a partner and/or children (N=800) | Sweden | Quantitative | Individual | Job demands increase work-to-family conflict, while a high degree of job control can reduce it | Connection between individuals’ psychosocial work environment and their capacity to strike a balance between paid work and family life. | 1(2); 2(1); 3(3) |
| **Hughes and Parkes (2007)** | To study mediating and moderating processes underlying the relationship between work hours and well-being. | 292 female employees in two UK public sector organizations | United Kingdom | Quantitative | Individual | Provision by employers of some flexibility and control over work hours may help to reduce the potential negative impact of long work hours | Insights from both labour market and organizational research could contribute to our understanding of how different aspects of work influence WFC | 1(1); 3(4); 4(1) |
| **Adam et al. (2008)** | To test the hypothesis that, compared to men, female physicians experience higher work—family conflict (WFC) and consequent burnout. | Hungarian physicians (N = 420) | Hungary | Quantitative | Individual | WFC emerged as a significant predictor of burnout (emotional exhaustion and depersonalization). | Female physicians scored significantly higher on the emotional exhaustion subscale of the Maslach Burnout Inventory and high levels of emotional exhaustion. | 1(3); 3(1); 6(1) |
| **Bakker et al. (2008)** | To explore integrative model delineating how job demands experienced by men and women carry over to the home domain. | Dual-earner parents (N=168) | Netherlands | Quantitative | Family | Hypothesis that gender would moderate the model relationships was rejected. | Theoretical model (he WFC–crossover model) can be used to integrate conflict and crossover theories. | 1(1); 4(4) |
| **Choi (2008)** | To understand the factors that cause life stress among employees. | Chinese employees (N=239) | China | Quantitative | Individual | Work and family demands cause life stress among Chinese employees both domains have a relationship with life stress to a similar extent | In a developing country family and work demands are related to employee stress and these demands have a relationship with life stress to a similar extent. | 1(1); 3(2); 4(1) |
| **Heponiemi et al. (2008)** | To explore perceptions of organizational fairness being able to diminish the negative effects of high job demands. | Women working in long‐term care for elderly people (N=713) | Finland | Quantitative | Individual | Organizational justice moderated the association between employment type and WIF | Promote fair organizational procedures and management in the health care sector especially for fixed-term employees or with fixed-term contracts. | 3(2); 4(1) |
| **Houkes et al. (2008)** | To examine specific patterns of relationships between job demands, job resources and person‐related factors. | Dutch General Practitioners GPs (N=700) Two waves | Netherlands | Qualitative Longitudinary | Individual | Pattern of relationships between job demands, job resources, person-related factors and burnout is different for men and women. | Anticipate in a gender sensitive way on risk factors for burnout and motivation loss for young professionals by coaching and empowerment in vocational training. | 1(1); 2(2); 3(1); 6(1) |
| **Ng and Feldman (2008)** | To examine the relationships between hours worked and indicators of organizational identity, occupational identity and family identity. | Studies Independent samples (N=222) | USA | Quantitative | Individual | Long work hours and family identification are generally only weakly related. | To get all the information of individuals’ motivation to work long hours, need to understand both the professional and situational contexts in which this dedication of time is made. | 3(4) |
|  |  |  |  |  |  | Long work hours do not necessarily yield significant gains in productivity. |  |  |
| **Bakker et al. (2009)** | To analyse the relationship between workaholism and relationship satisfaction. | Dual-earner couples (N=68) | Netherlands | Quantitative | Family | Workaholism is related to reduced support provided to the partner, through work-family conflict. | Workplace interventions as possible ways to help workaholics and their partners. | 1(1); 4(3); 6(1) |
|  |  |  |  |  |  | Gender did not affect the strength of the relationships in the proposed model. |  |  |
| **Kato and Yamazaki (2009)** | To explore relationship between work-related factors and work-to-family conflict and WFC, fatigue and depression. | Married Japanese employees chemical Industy (N=961) | Japan | Quantitative | Individual | WFC was unfavorably related to fatigue and depression in both genders regardless of parental status. | Strategies for reduction of WFC are therefore necessary to promote health among married workers of both genders. | 1(4); 5(1) |
|  |  |  |  |  |  | WFCplays a role linking unfavorable work situations and health. |  |  |
| **Cinamon and Rich (2010)** | To study interrelations between conflict and facilitation in work and family domains, with spousal, managerial, and collegial social support. | Female married teachers (N=322) | Israel | Quantitative | Individual | Managerial support predicted conflict and facilitation relations. Work-to-family and family-to-work conflict predicted burnout. | Conflict and facilitation are distinct constructs and they underscore the importance of working with managers to enhance their ability to promote employees’ health. | 1(2); 4(1); 5(1); 6(1) |
| **Fiksenbaum et al. (2010)** | To examine the relationship of work intensity and of work hours on potential antecedents and work and well‐being consequences. | Managers working hotels in Beijing (N=309) | China | Quantitative | Individual | The interaction of work intensity and work hours is not a significant predictor of work or well‐being outcomes. | Findings only partially consistent with previous conclusions suggesting the possible role played by cultural values and level of economic development. | 3(4) |
| **Siu et al. (2010)** | To test Western theories on work–family enrichment within the Chinese context. | Matched sample employees in China in two weves (N=786) | China | Quantitative | Individual | Work engagement was the most proximal predictor of work-family enrichment. | Encourage CEOs or human resource managers to provide more job resources in the workplace such as family-friendly organizational policies, supervisor support and job autonomy. | 2(4); 3(1); 5(1) |
| **Carlson et al. (2011)** | To explore organizational levers that impact work–family experiences, health, and subsequent turnover. | Women returning to full-time work 4 months after childbirth (N=179) | USA | Quantitative | Individual | Work-to-family conflict was negatively related to both physical and mental health | Impacts of job characteristics on WF mechanisms that play a role in the mental and physical health and retention of working mothers. | 1(3); 2(1); 3(1) |
|  |  |  |  |  |  | work-to-family enrichment positively predicted only physical health. |  |  |
| **Estryn-Behar et al. (2011)** | To explore how the organisation when working from home may be the heart of the problem for female and male physicians in France. | French hospital physicians (N=1924) | France | Quantitative | Individual | Excessive job demands are linked with burnout and with WFC,conducting to difficulties in organising one's life in order to have and raise children. | To facilitate teamwork to reduce departure, which increase workload on those who stay increasing their work family conflict. | 1(5); 6(1) |
| **Hakanen et al. (2011)** | To explore how job resources predict work engagement through WFE. | Two-wave, 3 years, Finnish dental Association (N= 1632) | Finland | Quantitative | Organizational | Results supporting the Conservation of Resources theory. | Study suggest that adding the idea of positive feedback loops might benefit the models theorizing WFE | 2(5); 4(1); 5(1) |
|  |  |  |  |  |  | Enrichment processes may take place among both women and men. |  |  |
| **Lu et al. (2011)** | To analyse whether family mastery enhances work engagement in Chinese nurses based on the theory of work–family enrichment and the job demands-resources (JD-R) model. | Chinese female nurses (N=279) | China | Quantitative | Individual | Resource generated in family could directly help people stay engaged in the workplace, particularly under stressful working conditions | To expand the JD-R model of work engagement and bridged it with theory of work–family enrichment. | 1(1); 3(1); 5(2) |
| **Shimazu et al. (2011)** | To examine the impact of workaholism on employees and their partners’ work-family conflicts and psychological distress. | Japanese dual-earner couples  (N=994) | Japan | Quantitative | Family | Workaholics were more likely to experience work-to-family conflict and psychological distress compared to relaxed workers for both genders. | Integrate and expand previous findings on workaholism and the recently formulated spillover-crossover model. | 1(2); 3(1); 4(2); 6(1) |
| **Cole and Secret (2012)** | To analyse job demands and workplace culture variables associated with work–family conflict stress, in addition to workplace racial bias. | African American women in 16 Fortune 1000 companies (N=607) | USA | Quantitative | Individual | Married women who experienced a more subtle form of workplace racial bias reported more work–family conflict stress. | Outline a potential plan and action steps to increase social work presence in the areas of policy, practice and research. | 1(2); 4(2) |
| **Gordon et al. (2012)** | To investigate the antecedents and consequences of both WIC (work interferes with caregiving) and CIW (caregiving interferes with work). | Women (N=583) | USA | Quantitative | Individual | Demands and supports related to the caregiving role do not influence work-related role strain. | Research on WFC is extended to de growing population of middle-aged and older employer women with significant elder caregiving responsibilities. | 1(1); 2(2) |
| **Eek and Axmon (2013)** | To explore possible associations between different subjective and objective work factors and benefits. | Working parents (N=1562) | Sweden | Quantitative Cross- Sectional | Individual | Work place factors related to flexibility and attitude to parenthood appear to have the strongest effect on working parents’ subjective stress and wellbeing, while benefits appear to have less impact. | A positive attitude towards parenthood and a flexible work situation seems beneficial for the general wellbeing and work engagement among working parents. | 3(1); 4(3); 5(1) |
| **Bowen et al. (2014)** | To investigate occupation stress and job demand, control, and support factors. | Architects, civil engineers, quantity surveyors, and project and construction managers (N=676) | South Africa | Quantitative | Individual | Critical job stressors identified arer: the presence of work–life imbalance, the need to ‘prove’ oneself, hours worked, working to tight deadlines. | Applicability of the JDC–S model in explaining occupational stress in the South African construction industry. | 3(6) |
| **Dishon-Berkovits (2014)** | To explore the Conservation of Resources Theory as a theoretical framework to examine the relationship between work–family and the three dimensions of job burnout. | Employed women (N=292) | Israel | Quantitative | Individual | Cross-domain compensation reduces each facet of job burnout. | The effects of the analysed variables were additive rather than interactive. | 1(1); 2(2); 4(1); 6(1) |
|  |  |  |  |  |  | Work–family conflict contributes to various dimensions of job burnout. |  |  |
| **Eek and Axmon (2015)** | To explore whether an unequal distribution of responsibilities in the home is related to various health related outcomes among women. | Women living relationship, working at least 50% of full time (N=37) | Sweden | Quantitative | Individual | Unequal distribution of responsibility for household duties showed higher levels of perceived stress, fatigue and WFC. | Key to work towards greater gender equality at home to maintain women's employment rate increase without it having a negative effect on women’s health and well-being. | 1(4) |
|  |  |  |  |  |  | Unequal distribution of responsibilities increased odds for insufficient time for various forms of recovery, which may further contribute to an increased risk of poor health. |  |  |
| **Nohe et al. (2015)** | To study the relationship between work–family conflict and strain. | Meta-analytic path analyses studies (N=33) | Diferentes Countries | Quantitative | Individual | WIF predicted strain and strain predicted WIF. | WIF had a stronger effect on work-specific strain than did FIW, supporting the matching hypothesis rather than the cross-domain perspective. | 1(1); 4(1) |
|  |  |  |  |  |  | Similarly, FIW and strain were reciprocally related. |  |  |
| **Daderman and Basinska (2016)** | To use Hobfoll’s conservation of resources (CORs) theory to explain the role of various resources in the improvement of work conditions in the nursing profession. | Female registered nurses, live with a partner and/or have children (N=88) | Poland | Quantitative | Individual | WFC was moderately related to FWC. | Produced new knowledge by examining a constellation of job demands, work engagement and WFC, which reflect the management of personal resources. Also, WFC does not intensify turnover intentions. | 1(2); 2(1); 3(1); 4(1); 5(1) |
|  |  |  |  |  |  | Only high job demands and low vigour were associated with turnover intentions. |  |  |
|  |  |  |  |  |  | WFC was experienced more intensively than FWC. |  |  |
| **Mache et al. (2016)** | To analyse the connection between physicians’ job demands and resources. | Clinicians specialising (N=564) | Germany | Quantitative Cross- Sectional | Individual | Relationships between physicians’ work engagement and their job satisfaction as well as between job stress and work family conflict. | Verifies the associations between work engagement, work-family conflict, job demands and resources influencing employees’ satisfaction. | 2(1); 3(2); 4(1) |
|  |  |  |  |  |  | Perceived job stress moderated the effect of high job demands on work family conflict. |  |  |
| **Nilsen et al. (2016)** | To identify trajectories of mental health problems from 1993 to 2006 in women. | Employed mothers drawn from the Tracking Opportunities and Problems-Study (TOPP) longitudinal study following Norwegian families across 18 years (N=439) | Norway | Quantitative Longitudinal | Family | High and Moderate symptoms as well as Low-Rising symptoms from 1993 to 2006 predicted higher levels of exhaustion, disengagement from work and work-family conflict in 2011. | Expand upon previous research by using a longer time span and focusing on employed women with children who experience different patterns of mental health trajectories. Highlight and validate the importance of early identification and prevention in women experiencing adverse patterns of mental health problems. | 1(3); 4(1); 6(1) |
| **Shukri et al. (2016)** | To study roles of work factors, work–family conflicts and culture on predictors of healthy intentions within the framework of the theory of planned behaviour (TPB). | Employees United Kingdom (N=278); Malaysia (N=325) | Malaysia, United Kingdom | Quantitative | Individual | Higher job demands were significantly related to lower intentions to eat a low‐fat diet. | Verified the efficacy of TPB variables in explaining intentions, with perceived behavioural control. Confirmed that TPB variables mediate the effects of job demands and job resources on intentions. | 1(4) |
|  |  |  |  |  |  | Women reported higher intentions to eat a low-fat diet than men. |  |  |
|  |  |  |  |  |  | UK participants had lower intentions to engage in physical activity vs. those from Malaysia. |  |  |
| **Milner et al. (2017)** | To analyse the relationship between working conditions and self-rated health among medical doctors in Australia. | Seven annual waves Medicine in Australia: Balancing Employment and Life (MABEL) survey (Doctors) | Australia | Quantitative | Individual | Excessive job demands, low job control, feelings of not being rewarded at work, and work-life imbalance were associated with higher within-person odds of poorer self-rated health. | The importance of addressing adverse working environments among doctors. | 1(3); 3(2) |
|  |  |  |  |  |  | For female doctors, work arrangements and work-life imbalance were associated with poorer self-rated health whilst task-based job stressors were associated with poorer self-rated health in male doctors. |  |  |
| **Oshio et al. (2017)** | To explore the mediating effect of work-to-family conflict (WFC) on the associations between eight types of job stressors. | Workers participated at three consecutive waves of J-HOPE (Japanese Study of Health, Occupation, and Psychosocial Factors Related Equity) Men (N=5859) Women (N=1560) | Japan | Quantitative | Individual | The mediating effect of WFC was smaller for job stressors indicating reduced job resources, compared with job demands and effort. | Policy measures and support from supervisors, to prevent job stressors from adding to WFC, are needed to reduce employee psychological distress. Not gender differences. | 1(1); 2(1); 3(4) |
|  |  |  |  |  |  | The mediating effect of WFC was larger for women than for men. |  |  |
| **Beauregard et al. (2018)** | To examine gendered pathways to burnout. | SALVEO study, a cross-sectional study of workers (N=2026 ) | Canada | Quatitative Cross-Sectional | Individual | Gender distinctively shapes environmental and individual pathways to burnout. | OHS prevention efforts striving for better mental health outcomes in the workforce could relevantly be informed by a gendered approach to burnout. | 1(2); 4(1); 6(1) |
| **Halliday et al. (2018)** | To develop and test a moderated mediation of the effects of perceived job autonomy on work–life balance, engagement, and turnover intentions, depending on FTE gender and country‐level gender egalitarianism (GE). | Workers (N=23439) | 26 Countries | Quantitative | Individual | Perceived job autonomy on work–life balance, engagement, and turnover intentions is stronger for women in lower GE countries and are not significant for men. | Discuss practical implications for engaging and retaining global female talent. | 1(4); 2(1); 4(1); 5(1) |
| **Lapierre et al. (2018)** | To examine theoretically derived antecedents of both directions of work–family enrichment. | Correlations (N=767) from independent studies (N=171) | N.A. | Quantitative Longitudinal | N.A. | Work engagement mediates between several contextual characteristics and enrichment is largely generalizable across populations. | Little evidence of gender being a moderator of relationships between contextual characteristics and enrichment. | 2(1); 4(1) |
| **Eguchi et al. (2019)** | To validate the Japanese version of the family supportive supervisor behaviours (FSSB-J) measure. | Married workers (N=1670) | Japan | Quatitative | Individual | FSSB-J is an adequate measure of FSSB in the Japanese context. | Sample was predominantly male (∼70% ), potentially limiting the generalizability of the current findings across genders. | 4(3) |
| **El-Kot et al. (2019)** | To explore the relation between perceived supervisor empowerment behaviours and feelings of personal empowerment. | Egyptian women managers and professionals (N=155) | Egypt | Quatitative | Individual | Perceived levels of supervisory/leader empowerment behaviours and self-reported feelings of empowerment had significant relationships with the majority of work and well-being outcomes. | A number of ways to increase levels of empowerment of both front-line employees and managers have been identified. | 3(1); 5(1) |
| **Minamizono et al. (2019)** | To study how gender division of labour affects the intention to leave the workplace among the nursing profession. | Female nurses (N= 328) | Japan | Quatitative | Individual | Above 70% of participants agreed with the gender division of labour that men should be the breadwinner and women should assume family responsibilities. | Young nurses and those who scored high on burnout were the most vulnerable population. | 1(4); 3(2); 6(1); |
|  |  |  |  |  |  | For young nurses, each domain of burnout score increased a risk of intention to leave, and high support decreased the risk. |  |  |
| **Gu et al. (2020)** | To analyse job demands, job resources, and emotional labour as antecedents of work-to-family conflict. | Female preschool teachers (N=320) | China | Quatitative | Individual | Job demands and surface acting led to an increase in work-to-family conflict over time. | Contribute to prior literature and show how the unfavourable effects of job demands and emotional labour may be avoided in the family domain through enhancing supervisor work–family support and fostering meaning of work. | 1(1); 2(4); 3(1) |
|  |  |  |  |  |  | Supervisor work–family support as well as deep acting directly reduced work-to-family conflict over time. |  |  |
|  |  |  |  |  |  | Meaning of work protected against increased work-to-family conflict. |  |  |
| **Rajendran et al. (2020)** | To study correlation of turnover intent among teachers examined through the lens of the job demands-resources (JD-R) model. | Teachers Primary (N=580) Secondary (N=675), male (N=254) and female (N=999) | Australia | Quatitative | Individual | Job demands and the personal demand of work–family conflict, were positively associated with emotional exhaustion, the core dimension of burnout. | JD-R is a promising theory for use in explaining job-related outcomes among Australian teachers. | 1(4); 2(1) |
|  |  |  |  |  |  | Work–family conflict was the strongest predictor of emotional exhaustion for male and female teachers. |  |  |
| **Zhang et al. (2020)** | To study work-family conflict´s relation with anxiety symptoms. | Female nurses and physicians (N=764) | China | Quantitative | Individual | The relationship between WFC and anxiety symptoms was weakest for female medical staff who reported high levels of social support. | Enrich the knowledge on mediating mechanisms, explaining associations between work-family conflict and anxiety symptoms. | 1(6) |
| **Abusanad et al. (2021)** | To examinee prevalence, work and lifestyle factors affecting Burnout (BO) | Oncology professionals (N=1017) | MENA | Qualitative | Invidual | BO was significantly associated with age <44y, administrative work >25% per day and the thought of quitting oncology (TQ) | Largest BO study in MENA. Quitting oncology is a simple and reliable screening tool for burnout. | 1(3); 6(2) |
| **Carlson et al. (2021)** | To examine the role of friend support in the work-family interface of dual-career couples. | Dual-earner married couples (N=76) | USA | Quantitative Longitudinal | Family | For men and women friend support played a critical role in reducing the resource depletion that contributed to the experience of work-to-family conflict. When considering crossover effects, the results differ by gender. | Developed a model that highlighted the potential role that friend support plays in reducing work-to-family conflict and diminished well-being associated with engagement at work | 1(3); 5(1) |
| **Chela-Alvarez et al. (2021)** | To explore in-depth factors perceived as stressors by hotel housekeepers (HHs). | Hotel Housekeepers (N=34) | Spain | Qualitative | Individual | HHs perceive their job to be physically and psychologically demanding and stressful. | To qualitatively explore, in-depth, the experience of hotel housekeepers in their job under the framework of the JD-R model and from a gender perspective. | 1(2); 2(1); 3(3); 4(1) |
|  |  |  |  |  |  | Work-home conflict is a relevant additional factor increasing the level of stress of HHs. |  |  |
| **Costantini et al. (2021)** | To analyse work–family policies related to work–family conflict as well as work–related attitudes among women in management positions returning to work after maternity leave. | Women executives (N=238) | Italy | Quantitative | Individual | Availability of policies was either directly or indirectly positively related to work attitudes among female managers. | Awareness of the available policies might be an important determinant of work-related well-being and organizational commitment. | 1(1); 5(3) |
| **Herman and Larouche (2021)** | To analyse associations between work/school commuting mode and multiple indices of subjective well-being and work-life balance. | (N = 7646) | Canada | Quantitative | Individual | Active commuting to work or school is positively associated with certain indices of subjective well-being and work-life balance among Canadians, especially among women. | Uniquely explored work-life balance and workaholism, using a large representative Canadian population sample. | 1(2); 6(1) |
| **Huml et al. (2020)** | To explore the relationship between work engagement and workaholism in sport employees. | Sport employees (N=4167) | USA | Quantitative | Individual | Significant, positive relationship between work engagement and workaholism. | To provide empirical support for a relationship previously conceptualized but untested within the business literature. | 1(1); 5(1); 6(2) |
|  |  |  |  |  |  | WFC may deter highly engaged workers from turning into workaholics. |  |  |
|  |  |  |  |  |  | Job flexibility and gender correlated with their reported levels of work engagement and workaholism. |  |  |
| **Janzen and Hellsten (2021)** | To study the socioeconomic disparities in mental health that were observed in a sample of employed, partnered mothers. | Employed partnered mothers (N=512) | Canada | Quantitative Cross- Sectional | Individual | Work–family conflict and inequity in the division of household labor may partially explain how lower income translates into greater psychological distress for employed partnered mothers. | Longitudinal research with diverse samples of women is required to elucidate how SES may differentially expose employed partnered mothers to hazards and resources within paid and domestic work contexts. | 1(2); 3(1) |
| **Lee et al. (2021)** | To illustrate how medical faculty’s experiences of their job demands and job resources affects their health statusand the role that the work-life interface plays in it. | Medical faculty top research hospital (N=30) | Canada | Qualitative | Organizational | Faculty’s job creates stress, work-life conflict, and exhaustion. | The precise nature and effects of job demands and job resources may be more complex than current research suggests. The use of thematic analysis allowed the researchers to better interpret the data. | 1(2); 2(1); 3(1); 5(1); 6(1) |
|  |  |  |  |  |  | Supportive job resources helped to mitigate this, yet stimulating job resources accentuated it. |  |  |
| **Pien et al. (2021)** | To study the effect of WFCs on the health status and nurses’ leaving intentions in Taiwan. | Nurses Regional Hospital (N= 200) | Taiwan | Quantitative | Individual | High levels of WFCs were associated with poor self-rated health and depression | For nurses, the high workload and inadequate family-friendly workplace policies and practices could be the root cause of WFCs, poor health, depression, and their intention to leave. | 1(4) |
|  |  |  |  |  |  | High levels of WFCs not associated with high leaving intentions. |  |  |
| **Pujol-Cols (2021)** | To develop a Spanish version of the Work-Family Conflict Scale (SP-WFCS). | Two independent samples Argentinian employees (*N*=618) | Argentina | Quatitative | Individual | The six-dimensional model of the SP-WFCS was statistically invariant across samples and gender. | Provided support to the validity of the SP-WFCS in Argentina, suggesting that it may be a reliable instrument to measure work-family conflict in Spanish-speaking countries. | 1(1); 2(1); 3(1); 6(1) |
|  |  |  |  |  |  | Most work-family conflict dimensions displayed correlations with quantitative and emotional demands and core self-evaluation as well as affective job satisfaction and burnout. |  |  |
| **Xu and Li (2021)** | To to translate the Multidimensional Workaholism Scale (MWS) into Chinese and test its reliability and validity. | Chinese employees (N1=220) (N2=425) | China | Quantitative | Individual | Chinese version of the MWS is a valid and reliable tool for Chinese employees, | Chinese version of the MWS did not differ across gender, age, and job position groups. Workaholism and engagement correlated with emotional exhaustion, WFC and life well-being | 6(1) |
| **Adams and Golsch (2022)** | to Investigate the extent to which employed mothers and fathers scale back on working hours or job pressures in response to work-to-family conflicts (WFC). | Mothers (N=791) Fathers (N=1292) | Germany | Quantitative | Individual | Scaling back seems not to be a commonly used strategy to react to WFC. | Gender differences in the reaction between mothers and fathers on WFC only occur in connection with traditional gender ideology. | 1(2); 4(1) |
| **Ayar et al. (2022)** | To study how demographical and individual factors and work addiction impact work-life balance. | Health Professionals (N=336) | Turkey | Quantitative | Individual | Gender, working hours, work addiction, and PTLW had direct effects on mental health and WLB in the final model. | Work-life balance can be explained by variables such as gender, work addiction, working hours, and the people with whom health professionals live during the pandemic. | 1(1); 2(1); 4(1); 6(1) |
| **Bradfield et al. (2022)** | To explore the association between medical negligence claims and doctors’ sex, age, specialty, working hours, work location, personality, social supports and family circumstances. | Australian doctors (N=12134) | Australia | Qualitative | Individual | Modifiable risk factors contribute to an increased risk of medical negligence claims among doctors in Australia. | Working in a regional area, low self-rated life satisfaction and not achieving work–life balance predicted medical negligence claims in male, but not female, doctors. HWorking more than part-time hours and having a recent personal injury or illness predicted medical negligence claims in female, but not male, doctors. | 1(2) |
| **Eason et al. (2022)** | To examine the effect that work addiction may have on work-life interface outcomes in athletic training. | Athletic trainers (N=226) | USA | Quantitative Cross- Sectional | Individual | ATs at higher risk for work addiction reported higher levels of burnout. | Women were more at risk for compulsive tendencies than were men. | 1(1); 6(4) |
| **Falco et al. (2022)** | To confirm validation of the Bergen Work Addiction Scale (BWAS) in the Italian context. | Bank workers (N= 8419) | Italy | Quantitative | Organization | BWAS-I showed adequate psychometric properties in terms of factor structure, reliability and measurement invariance across gender and managerial status. | Some items intercepts were not equivalent across gender and managerial status | 1(1); 3(1); 6(1) |
| **Innstrand and Grodal (2022)** | To study antecedents, consequences, and potential gender differences of perceived inclusion, a new concept salient to contemporary work life. | Academics and faculty staff higher education institutions Norway (N=12170) | Norway | Quantitative | Individual | Model were stronger for men than women. | Inclusion was measured with only surface-level diversity characteristics (gender, age, disability, and ethnicity). | 1(3); 4(2) |
|  |  |  |  |  |  | Not only do men perceive their work environment as more inclusive. |  |  |
|  |  |  |  |  |  | Is also more strongly related to beneficial outcomes for the organization. |  |  |
| **Jolly et al. (2022)** | To analyse how family-supportive supervisor behaviours (FSSB) influence employee emotional exhaustion (EE). | Foodservice employees (N=226) | USA | N.A. | Individual | FSSB decreased employee EE and subsequent turnover intentions. This relationship was stronger for female employees. | Women still shoulder a majority of home and non-work caring duties. They may be more positively affected when supervisors engage in behaviours that allow for balancing work and non-work demands. | 1(4); 3(1); 4(1) |
| **Lange and Kayser (2022)** | To analyse the relation between self-efficacy (SE), work-related stress (WRS) and health outcomes (health and anxiety) in a remote work setting. | German remote work population (N =5163) | Germany | Quantitative | Individual | Importance of self-efficacy as a personal resource to buffer WRS and WFC while promoting overall health at the same time. | Future studies may also analyze interventions that promote self-efficacy for specific target groups such as supervisors and managers or women and men. | 1(2); 2(3); 3(1) |
| **Lyu and Fan (2022)** | To examine the role of job-crafting and gender on the work-family conflict. | N.A. | China | N.A. | N.A. | There was no significant difference between men and women in work-family conflict. | Help to understand the impact of gender on work-family interface, and provide valuable implications for the organizational strategies and social support systems to deal with work-family conflict. | 1(2); 5(1) |
|  |  |  |  |  |  | Participants would not lower their work engagement when work interfered with family issues. However, when families interfered with work, women were becoming less likely to engage in work. |  |  |
| **Orfei et al. (2022)** | To adapt demands on a broad population of employees of a large Italian banking group in the job-related stress framework. | Bank Employees in three waves (N1=1264, N2=841, N3= 491) | Italy | Quantitative | Organizational | Massive adjustment demands in work and family routine represented a significant source of stress for employees, regardless of the different pandemic stages. | After a long and stressful period of work-family routine adjustment, women best appreciate HW’s advantages and are less prone to BW. | 1(1); 2(2); 3(1) |
| **Saleem et al. (2022)** | To examine the role of emotional labour for university teachers in Pakistan, developing an Employee Emotional Engagement Model in the work and family domain | Teachers University COMSATS Islamabad (N= 126) | Pakistan | Quantitative | Organizational | Females experienced stronger enrichment from work to family while deep acting compared to males. | Work-to-family conflict has negative and work-to-family enrichment has a positive relationship with family engagement, but do not show any difference between males and females. | 1(1); 4(1); 5(2) |
|  |  |  |  |  |  | Family engagement strongly predicts work engagement in employees. |  |  |
| **Scheibe et al. (2022)** | To examine the relation between age, resilience, job demands and resources, and self-regulation, during the COVID-19 pandemic. | Employees Dutch University (N=1715) | Netherlands | Quantitative/Qualitative) | Individual | Age was related to resilience during the COVID-19 pandemic, with higher mental, cognitive, and social well-being levels being related to advanced age | Robust even after controlling for influential background factors, such as gender, expat status, job type, and living alone. | 2(4); 3(1) |
| **Syrek et al. (2022)** | To explore the effects of a new leave policy that was introduced in a large Dutch Financial Company. | Enployees (N=537) | Netherlands | Quantitative Longitudinal | Organizational | Work-related well-being indicators are differently affected by the pandemic. | Pre-pandemic longitudinal data collection affords the researchers to have a rare view of the developments in employees' work-related well-being during the first weeks of the crisis as well as in the months after the onset of the crisis. | 2(4); 4(1) |
|  |  |  |  |  |  | Women and younger workers are more seriously affected by the crisis. |  |  |
| **Vitoria et al. (2022)** | To review the literature addressing the COVID-19 impact on the work-family interface. | Studies (N= 32) | Diferents countries | Theoretical: sistematic revue | N.A. | The COVID-19 crisis has affected the work-family interface mainly due to the need to promptly adapt to remote work, greater unpaid and paid workload, and higher role strain. | The COVID-19 outbreak has pushed men and women to perform traditional roles. | 1(1); 2(2); 4(2) |
| **Zhang and Wang (2022)** | To examine the lasting effect of reproductive behaviour on career cycles for women. | Working mothers with two or more children (N= 306) | China | Quantitative | Individual | Chain mediation variables of family orientation (FO) and work engagement (WE) mediated the relationship between FWC and EE significantly, as well as between HCD and EE. | In the context of fertility policy adjustment and population structural imbalance, research from the perspective of females may better reflect reality and deepen understanding of EE to avoid it. | 1(1); 4(1); 5(1) |
| **Chen et al. (2023)** | To evaluate work-to-family conflict´s (WFC) influence on unethical pro-family behaviour (UPFB) and work engagement (WE). | Chinese female leaders (N/A) | China | Quantitative | Individual | Significant positive correlation with UPFB and a negative correlation with WE | Examines Chinese female leaders in organizations, their current experience of WFC and the resulting psychological and behavioural outcomes. | 1(1); 5(4) |
|  |  |  |  |  |  | WFG with a mediating role in the relationships between WFC and both UPFB and WE |  |  |
|  |  |  |  |  |  | Significant moderating effect of FC on the relationship between WFC and WE |  |  |
| **De Beer et al. (2023)** | To study validity, reliability, and measurement invariance of the original BAT-23 and shortened BAT-12 in Norway. | Employees (N= 493) | Norway | Quantitative | Individual | Burnout acts as a mediator in our proposed job demands–resources model as preliminary evidence of predictive validity. | Reinforce the importance of job demands and resources, along with burnout as a key mediator, in understanding workplace dynamics in accordance with job demands–resources theory | 2(1); 3(1); 6(1) |
| **Dishon-Berkovits et al. (2023)** | To build on job demands-resources (J-DR) and self-determination (SDT) theories to investigate differences across the life course in the effect of communion job resources and job hindrance demands. | 6th European Working Conditions Survey Employees (N= 35377) | 35 Countries | Quantitative | Individual | Communion job resources are associated with lower WFC, which in turn is associated with enhanced wellbeing. | The model is significant for both men and women from all age groups, but parameter estimates magnitude are stronger for women aged 50 + . | 1(1); 2(2); 3(1) |
|  |  |  |  |  |  | Communion job hindrance demands and structural demands are related to heightened WFC, which is translated into diminished wellbeing. |  |  |
| **Lai and Lee (2023)** | To analyse the intrinsic mechanism of female leaders’ occupational stigma consciousness regarding career development and work–life balance. | Chinese female leaders (N=400) | China | Quantitative | Individual | Stigma consciousness has a significant negative impact on career development and work–life balance. | The stigma consciousness of female leadership has a significant negative impact on their career advancement and work–life balance. This negative influence is mediated by emotional exhaustion | 4(1); 5(1) |
|  |  |  |  |  |  | Emotional exhaustion plays a mediating role and self-efficacy negatively moderates the relationship between female leadership stigma consciousness and emotional exhaustion. |  |  |
| **Lu (2023)** | To explore the relation between children's depressive moods and parental family-work interaction. | Adolescent-parent, local senior high school (N = 468) | China | Quantitative | Family | Adolescents’ performance-avoidance orientation moderates the indirect relationship between adolescents' depressive moods and parents' work engagement *via* increased parents FWC | The depressive moods of their children's might make it worse or even bring about a vicious circle of work-family interaction. Women may face more severe challenges in 24/7 work culture. | 1(1); 4(1); 5(1) |
| **Lv et al. (2023)** | To examine the dual-squeeze effect of professional life on personal life. | N.A. | Asia | Quantitative | Individual | All three types of job demands (time demand, work intensity and emotional labour demand) are positively associated with task performance | The impacts of overwork on family satisfaction vary with marital status and gender | 1(1); 2(1) |
| **Mashaba and Botha (2023)** | To investigate the influence of women's engagement in technical positions in the South African mining sector. | Women in technical mining positions (N= 282) | South Africa | Quantitative / Qualitative | Individual | Importance of self-efficacy as a personal resource to buffer WRS and WFC while promoting overall health at the same time. | Mining organizations should accord high priority to enhancing working conditions for women and adopting work arrangements that foster a healthy work-life balance. | 1(2); 4(1); 5(1) |
| **Milicev et al. (2023)** | To investigate the prevalence and provenance of anxiety, depression, sleep problems, subjective mental wellbeing, and suicide behaviours in the United Kingdom. | Postgraduate research (PGRs) UK (N=479) | United Kingdom | Quantitative Longitudinal | Individual | Universities may aim to enhance PGR welfare by committing to the values of equality, inclusivity, and diversity. | Female gender was a risk factor for depression. Non-binary gender was linked to poorer levels of wellbeing. Non-heterosexual PGRs reported poorer sleep, lower wellbeing and an increased risk of suicide in comparison to heterosexual PGRs. | 1(5) |
|  |  |  |  |  |  | Promoting PGR integration, social support, and a healthy work-life balance. |  |  |
| **Olsen et al. (2023)** | To examine the relation between job demands and resources, and job stress and engagement, using the job demands–resources (JD-R) model, adapted to working from home during the pandemic. | Employees Telecommunications Company (N = 303) | Norway | Quantitative | Organizational | The difficulty of work tasks is positively related to stress while time spent working from home, | The option to work from home may reduce stress among these young employees, however working from home may face limitations regarding enhancing job engagement. | 2(3); 3(3); 5(1) |
|  |  |  |  |  |  | Support from leaders and colleagues and managing work–life balance are positively related to job engagement. |  |  |
| **Ratnaningsih et al. (2023)** | To study spill over/crossover effects on the work–family interface. | Teachers and their spouses (N = 600) | Indonesia | Quantitative | Family | Significant spillover–crossover effects of WFC on personal burnout amongst working wives, but not working husbands. | Exploring the crossover effects of WFC and FWC amongst marital spouses in a country with inherent conservatism and traditional gender role perspectives. | 1(1); 4(5); 6(1) |
| **Russo et al. (2023)** | To examine the workaholic–personal burnout relationship by using the Actor-Partner Interdependence Mode. | Italian couples dual -carreer (N=138) | Italy | Quantitative | Family | Levels of workaholism of partners are related and that for both partners. | The study has several strengths—such as the fact that it is based on multisource data and that it used the APIM model | 1(1); 4(2); 6(2) |
|  |  |  |  |  |  | Workaholism is related to burnout suggesting a very detrimental impact of workaholism in the life of working couples and their families. |  |  |
| **Tang et al. (2023)** | To explore the impact of work-to-family conflict (WFC) on work engagement | Chinese preschool teachers (N = 203) | China | N.A. | Individual | The relationship between WFC and work engagement was mediated by psychological detachment, explicitly and implicitly. | Gender could only moderate the relationship between WFC and explicit psychological detachment. | 1(4); 5(1) |
| **Tsang et al. (2023)** | To explore the relation between family–work conflict and work-from-home productivity based on role conflict and resource drain theories, as well as the family–work conflict literature. | Employees SME (N =785) | Taiwan | Quantitative | Individual / Organizational | The importance of psychological factors when it comes to explaining the impact of family–work conflict on WFHP during the pandemic. | This study proposes a new model to investigate the association between FWC and WFHP in Taiwan during Covid-19. | 1(3); 2(4) |
